# The diadenosine tetraphosphate hydrolase ApaH contributes to *Pseudomonas aeruginosa* pathogenicity

**DOI:** 10.1371/journal.ppat.1012486

**Published:** 2024-08-19

**Authors:** Matteo Cervoni, Davide Sposato, Giulia Ferri, Heike Bähre, Livia Leoni, Giordano Rampioni, Paolo Visca, Antonio Recchiuti, Francesco Imperi

**Affiliations:** 1 Department of Science, University Roma Tre, Rome, Italy; 2 Department of Medical, Oral and Biotechnology Sciences, University of Chieti-Pescara, Chieti, Italy; 3 Research Core Unit Metabolomics, Hannover Medical School, Hannover, Germany; 4 IRCCS Fondazione Santa Lucia, Rome, Italy; 5 NBFC, National Biodiversity Future Center, Palermo, Italy; University of Maryland, UNITED STATES OF AMERICA

## Abstract

The opportunistic bacterial pathogen *Pseudomonas aeruginosa* causes a wide range of infections that are difficult to treat, largely because of the spread of antibiotic-resistant isolates. Antivirulence therapy, *í*.*e*. the use of drugs that inhibit the expression or activity of virulence factors, is currently considered an attractive strategy to reduce *P*. *aeruginosa* pathogenicity and complement antibiotic treatments. Because of the multifactorial nature of *P*. *aeruginosa* virulence and the broad arsenal of virulence factors this bacterium can produce, the regulatory networks that control the expression of multiple virulence traits have been extensively explored as potential targets for antivirulence drug development. The intracellular signaling molecule diadenosine tetraphosphate (Ap4A) has been reported to control stress resistance and virulence-related traits in some bacteria, but its role has not been investigated in *P*. *aeruginosa* so far. To fill this gap, we generated a mutant of the reference strain *P*. *aeruginosa* PAO1 that lacks the Ap4A-hydrolysing enzyme ApaH and, consequently, accumulates high intracellular levels of Ap4A. Phenotypic and transcriptomic analyses revealed that the lack of ApaH causes a drastic reduction in the expression of several virulence factors, including extracellular proteases, elastases, siderophores, and quorum sensing signal molecules. Accordingly, infection assays in plant and animal models demonstrated that ApaH-deficient cells are significantly impaired in infectivity and persistence in different hosts, including mice. Finally, deletion of *apaH* in *P*. *aeruginosa* clinical isolates demonstrated that the positive effect of ApaH on the production of virulence-related traits and on infectivity is conserved in *P*. *aeruginosa*. This study provides the first evidence that the Ap4A-hydrolysing enzyme ApaH is important for *P*. *aeruginosa* virulence, highlighting this protein as a novel potential target for antivirulence therapies against *P*. *aeruginosa*.

## Author summary

Novel therapeutic interventions are urgently needed to address the threat of antimicrobial resistance. Inhibition of the virulence potential of bacterial pathogens, the so called antivirulence approach, is an alternative or complementary strategy to standard antibiotic-based therapies. The identification of suitable targets is essential to promote and advance antivirulence drug discovery programs. By combining *in vitro* analyses and *in vivo* infection assays, we demonstrated that the enzyme ApaH, which is responsible for the degradation of the intracellular signaling molecule diadenosine tetraphosphate, contributes to the virulence of the important human pathogen *Pseudomonas aeruginosa*. Indeed, loss of ApaH drastically reduces the production of several virulence factors in both laboratory and clinical isolates, and impairs *P*. *aeruginosa* ability to cause infection in plant, insect and rodent models. These results, together with the absence of ApaH homologues in mammals, make ApaH a promising target for the development of anti-*P*. *aeruginosa* antivirulence drugs.

## Introduction

*Pseudomonas aeruginosa* is a Gram-negative bacterium widely studied for its pathogenic nature, particularly in the context of opportunistic infections in hospitalized, immunocompromised and cystic fibrosis (CF) individuals [1]. A recent study associated *P*. *aeruginosa* with more than 500,000 deaths in 2019 globally [2]. The bacterium is well known for its metabolic plasticity, capability to thrive in many different environments, large arsenal of virulence factors, and resistance to multiple antibiotics [3–5]. The latter is due to inherent low membrane permeability and expression of several efflux pumps, as well as to the ability to readily acquire new resistance determinants via mutations or horizontal gene transfer [6,7].

Successfully thriving in diverse environments requires complex regulatory networks that help bacterial cells perceive the environment and adapt their behavior. *P*. *aeruginosa* has a large genome (6.3 Mb for the reference strain PAO1), 10% of which is dedicated to regulatory networks, reflecting its high versatility and the wide range of environments and hosts it can inhabit [8]. The contribution of quorum sensing (QS) systems, two-component systems, and nucleotide-based second messengers to *P*. *aeruginosa* stress response, metabolic versatility, switch from planktonic to biofilm lifestyle, and virulence gene regulation has been extensively investigated [3,9–11]. Concerning the roles of nucleotide-based second messengers in *P*. *aeruginosa*, the stringent response alarmone guanosine tetraphosphate (ppGpp) promotes survival under nutrient starvation and antibiotic tolerance [12], cyclic di-GMP (c-di-GMP) is the primary intracellular signal that controls biofilm development [13], while cyclic AMP triggers virulence gene expression in response to surface sensing [14]. Notably, to date the role of another widely distributed nucleotide-based second messenger, *i*.*e* diadenosine tetraphosphate (Ap4A), has not been explored in this bacterium.

Ap4A (or Ap3A) has been found in all kingdoms of life and consists of two adenosines linked by a polyphosphate chain containing four (or three) phosphates [15,16]. Ap4A is primarily generated as a side product of aminoacyl-tRNA synthetase activity [17] and its levels increase in response to several types of stress [18]. Ap4A intracellular homeostasis relies on the activity of Ap4A-hydrolyzing enzymes, such as the diadenosine tetraphosphatase ApaH or members of the versatile family of Nudix hydrolases [18–20]. ApaH appears to play a prominent role in Ap4A homeostasis in Gram-negative bacteria [18,21]. Since its discovery decades ago, there has been debate about the function of Ap4A, leading to the alternative hypotheses that Ap4A may be either a damage metabolite or an intracellular signaling molecule [18,22].

Several studies have demonstrated that Ap4A has pleiotropic effects in bacteria. In the model organism *Escherichia coli*, Ap4A has been found to affect the timing of cell division [23], repress motility [24], increase sensitivity to heat and oxidative stress [25], and reduce tolerance to aminoglycoside antibiotics [26,27]. Regarding other bacteria, Ap4A has a negative impact on sporulation in *Myxococcus xanthus* [28], reduces oxidative stress resistance in *Helicobacter pylori* [29], and limits invasion of mammalian cells by *Salmonella enterica* [21], while it promotes biofilm formation in *Pseudomonas fluorescens* [30].

By combining genetic, transcriptomic, and phenotypic *in vitro* analyses with *in vivo* infection assays, in this study we demonstrate that ApaH inactivation in *P*. *aeruginosa* leads to intracellular accumulation of Ap4A and drastically reduces virulence gene expression and pathogenicity. This work paves the way for the investigation of regulatory mechanisms underlying Ap4A-mediated control of *P*. *aeruginosa* virulence and for the exploitation of ApaH as a novel potential target for antivirulence drug discovery.

## Results and discussion

### Effect of Ap4A accumulation on *P*. *aeruginosa* growth and biofilm formation

**Fig 1:**
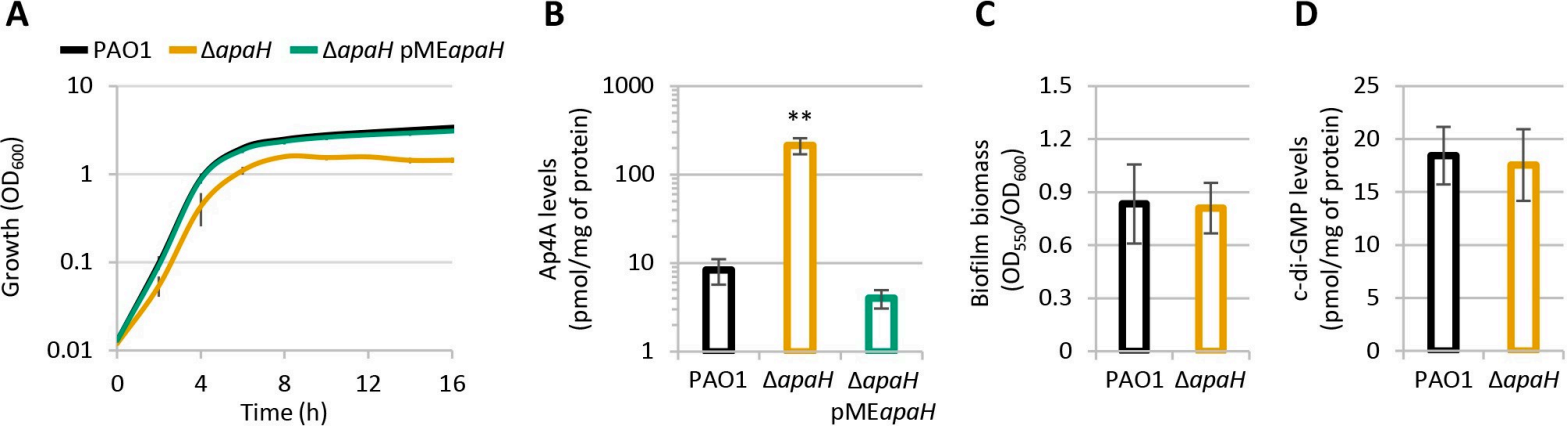
(**A**) Growth curves and (**B**) intracellular Ap4A levels of the wild type strain *P*. *aeruginosa* PAO1 and the isogenic deletion mutant Δ*apaH*, carrying or not the pME*apaH* plasmid, cultured in flasks at 37°C in LB, supplemented with 100 μM IPTG in the case of Δ*apaH* pME*apaH*. The control strains with the empty plasmid pME6032 are shown in S1 Fig. (**C**) Biofilm formation of PAO1 and Δ*apaH* in LB in 96-well polystyrene microtiter plates after 24-h incubation at 37°C. (**D**) Intracellular c-di-GMP levels of PAO1 and Δ*apaH* cultured in LB at 37°C. Cells for Ap4A and c-di-GMP quantification were collected after 12 h of growth. Values are the mean (± standard deviation) of at least three independent experiments. Asterisks indicate a statistically significant difference (*P* < 0.001) with respect to PAO1 (ANOVA).

In order to verify the influence of Ap4A in *P*. *aeruginosa* physiology, we generated a clean *apaH* deletion mutant in the reference strain PAO1. Growth assays in the rich medium Lysogeny broth (LB) showed that the *apaH* mutant has slightly reduced growth rates (doubling time of 37 and 40 minutes for the wild type and mutant, respectively) and anticipates the entry into the stationary phase, reaching cell densities 2.3-fold lower than the wild type strain (Fig 1A). Mass spectrometry analysis showed that Ap4A levels in cell extracts are 25-fold higher in the *apaH* mutant with respect to the wild type strain (Fig 1B). The ectopic expression of *apaH* from the pME*apaH* plasmid in the *apaH* mutant restored both growth and Ap4A intracellular concentration at wild type levels (Figs 1A, 1B and S1). It was reported that Ap4A promotes biofilm formation in *P*. *fluorescens*, in line with the increase in c-di-GMP intracellular levels observed in ApaH-deficient *P*. *fluorescens* cells [30]. We therefore investigated the impact of ApA4 on biofilm formation in *P*. *aeruginosa*. Surprisingly, we did not observe any difference in either biofilm formation or c-di-GMP intracellular levels between wild type and Δ*apaH* cells (Fig 1C and 1D). Overall, these experiments demonstrate that the lack of ApaH causes a marked increase in the Ap4A intracellular levels also in *P*. *aeruginosa* and that Ap4A accumulation has a negative impact on planktonic growth in this bacterium. In contrast, increased Ap4A levels do not appear to affect biofilm formation or c-di-GMP homeostasis in *P*. *aeruginosa*, suggesting that the cellular pathways affected by Ap4A may differ significantly also between closely related species. Notably, a slight 20% decrease in the ADP levels was observed in the Δ*apaH* mutant with respect to the wild type, while ATP levels were comparable between the two strains (S2 Fig). The effect of ApaH depletion on ADP levels seems to be specific, as no differences were observed in GDP and GTP levels (S2 Fig). This result is in line with the activity of ApaH as a symmetric diadenosine tetraphosphatase which cleaves Ap4A in two ADP molecules [19,20], and also suggests that Ap4A accumulation has a minor impact on the energy status of the cells.

### Ap4A does not affect *P*. *aeruginosa* susceptibility to antibiotics

**Fig 2:**
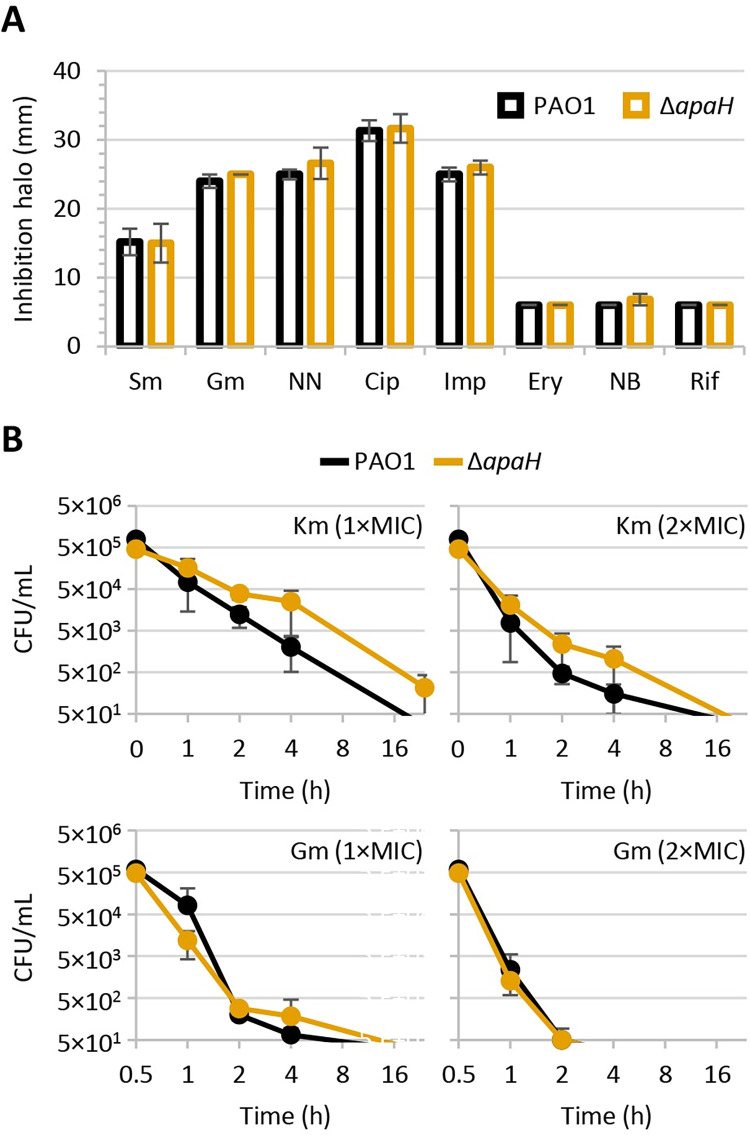
(**A**) Inhibition halos of streptomycin (Sm), gentamicin (Gm), tobramycin (NN), ciprofloxacin (Cip), imipenem (Imp), erythromycin (Ery), novobiocin (NB), or rifampicin (Rif) for *P*. *aeruginosa* PAO1 and the Δ*apaH* mutant in the Kirby-Bauer disc diffusion assay. (**B**) Time-kill curves of PAO1 and Δ*apaH* in the presence of kanamycin (Km) at 32 and 64 μg/mL or Gm at 0.5 and 1 μg/mL, corresponding to 1× and 2×MIC for both strains. Values are the mean (± standard deviation) of at least three independent assays.

Previous works showed that *apaH* inactivation in *P*. *aeruginosa* (i) slightly induces the expression of the colistin resistance gene *eptA*, without however affecting colistin resistance [31], and (ii) makes *P*. *aeruginosa* cells harboring an aminoglycoside resistance plasmid more susceptible to killing by millimolar concentrations of kanamycin [26]. To verify whether Ap4A has a general effect on antibiotic resistance in *P*. *aeruginosa*, the resistance profile of PAO1 and Δ*apaH* cells was compared through the Kirby-Bauer disc diffusion assay. As shown in Fig 2A, *apaH* inactivation and, thus, Ap4A accumulation does not increase sensitivity to any of the antibiotics tested, including clinically relevant antibiotics used to treat *P*. *aeruginosa* infections (*i*.*e*., ciprofloxacin, imipenem, tobramycin, gentamycin) or antibiotics that are only active against *P*. *aeruginosa* mutants with defects in cell envelope integrity (*i*.*e*., novobiocin, erythromycin, rifampicin) [32]. It was previously reported that Ap4A can enhance the bactericidal activity of aminoglycoside antibiotics in *E*. *coli* and, likely, other bacteria [26]. Therefore, we performed time-kill assays for two aminoglycosides, namely kanamycin and gentamicin. ApaH-deficient cells showed a slightly delayed killing by kanamycin with respect to wild type cells, while the susceptibility of the wild type and Δ*apaH* strains to gentamycin killing was comparable (Fig 2B). Overall, these results argue against a relevant contribution of Ap4A in intrinsic antibiotic resistance in *P*. *aeruginosa*. The discrepancy between our and previous results [26] regarding kanamycin sensitivity is possibly due to the different genetic backgrounds and experimental conditions, as we used the wild type strain PAO1, while Ji and coauthors used PAO1 carrying an aminoglycoside resistance plasmid challenged with a very high kanamycin concentration (1,000 μg/mL) [26].

### ApaH inactivation widely affects *P*. *aeruginosa* gene expression

To investigate the possible role of Ap4A in *P*. *aeruginosa* physiology, we compared the transcriptional profiles of wild type and Δ*apaH* cells cultured to mid-exponential phase in LB by means of RNA sequencing (RNA-seq). Following statistical validation of the dataset, 1,280 differentially regulated genes (DEGs) with a fold change (FC) ≥ ± 2.0 and an adjusted *P* value < 0.05 were identified (S1 Table), corresponding to 22.3% of the PAO1 genome (https://pseudomonas.com) [33]. Of these, 799 were downregulated in ApaH-deficient cells (S1 Table). This indicates that the rise in the Ap4A intracellular level caused by *apaH* deletion has a huge impact on the *P*. *aeruginosa* transcriptome, with most of the DEGs being repressed by Ap4A (62.4%). By applying more stringent thresholds (*i*.*e*., FC ≥ ± 3.0 or ≥ ± 4.0) the number of DEGs drops to 477 and 267, respectively (S1 Table), highlighting that Ap4A has only a moderate effect on the transcription of most DEGs. Considering that *apaH* deletion slightly affects growth (Fig 1A), it is likely that some of these DEGs are affected indirectly due to changes in the physiological and/or metabolic status of the cells upon AP4A intracellular accumulation.

**Fig 3:**
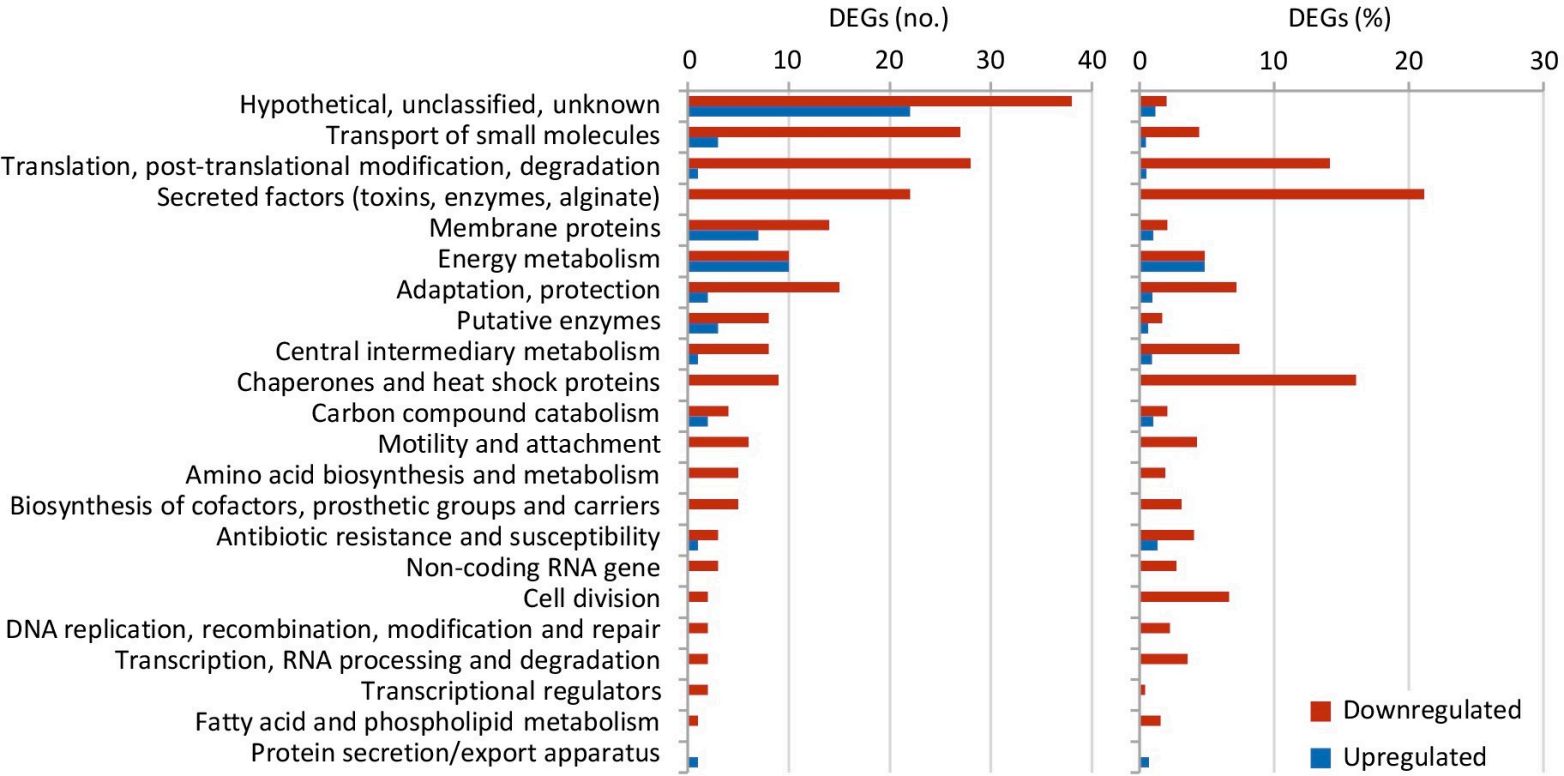
Functional analysis of differentially expressed genes (DEGs). Number (left panel) and relative percentage (right panel) of downregulated and upregulated genes (red and green bars, respectively) in the *apaH* mutant compared to PAO1 in the PseudoCAP functional categories listed on the left. Only DEGs with a fold change ≥ ± 4 were considered (S1 Table).

According to PseudoCAP annotations (https://pseudomonas.com), most of the highly affected DEGs (FC ≥ ± 4.0) are involved in virulence factors production, stress response, and translation (Fig 3). In particular, many genes important for *P*. *aeruginosa* pathogenicity were downregulated in ApaH-deficient cells, including those encoding extracellular proteases (*i*.*e*., AprA, LasA, LasB, and protease IV), pyocyanin biosynthetic enzymes, type IV pili, the adhesin LecA, and enzymes responsible for the synthesis of the *pqs* QS signal molecules (PQS and HHQ) (S1 Table). Moreover, genes involved in iron uptake (*e*.*g*., those responsible for the synthesis and transport of the two siderophores pyoverdine and pyochelin) were downregulated, while those involved in iron storage (*e*.*g*., bacterioferritin genes) were upregulated in the *apaH* mutant relative to PAO1 (S1 Table), suggesting that ApaH deficiency and/or Ap4A accumulation dysregulate intracellular iron homeostasis. Among DEGs involved in translation, it is worth noting that almost all genes encoding ribosomal proteins were downregulated in the *apaH* mutant (S1 Table), suggesting that Ap4A might have a negative impact on protein translation. In this view, it is surprising that ApaH-proficient and -deficient cells showed comparable susceptibility to inhibitors of protein synthesis, such as the aminoglycosides gentamicin, kanamycin, streptomycin, and tobramycin (Fig 2). While most DEGs were downregulated in the *apaH* mutant (53 upregulated *vs* 214 downregulated genes at the FC ≥ ± 4.0 threshold), energy metabolism was the only functional category for which the number of up- and downregulated DEGs was comparable (Fig 3 and S1 Table), suggesting that intracellular Ap4A accumulation may induce some metabolic shifts in *P*. *aeruginosa*.

### ApaH inactivation suppresses *P*. *aeruginosa* virulence traits and reduces heat tolerance

**Fig 4:**
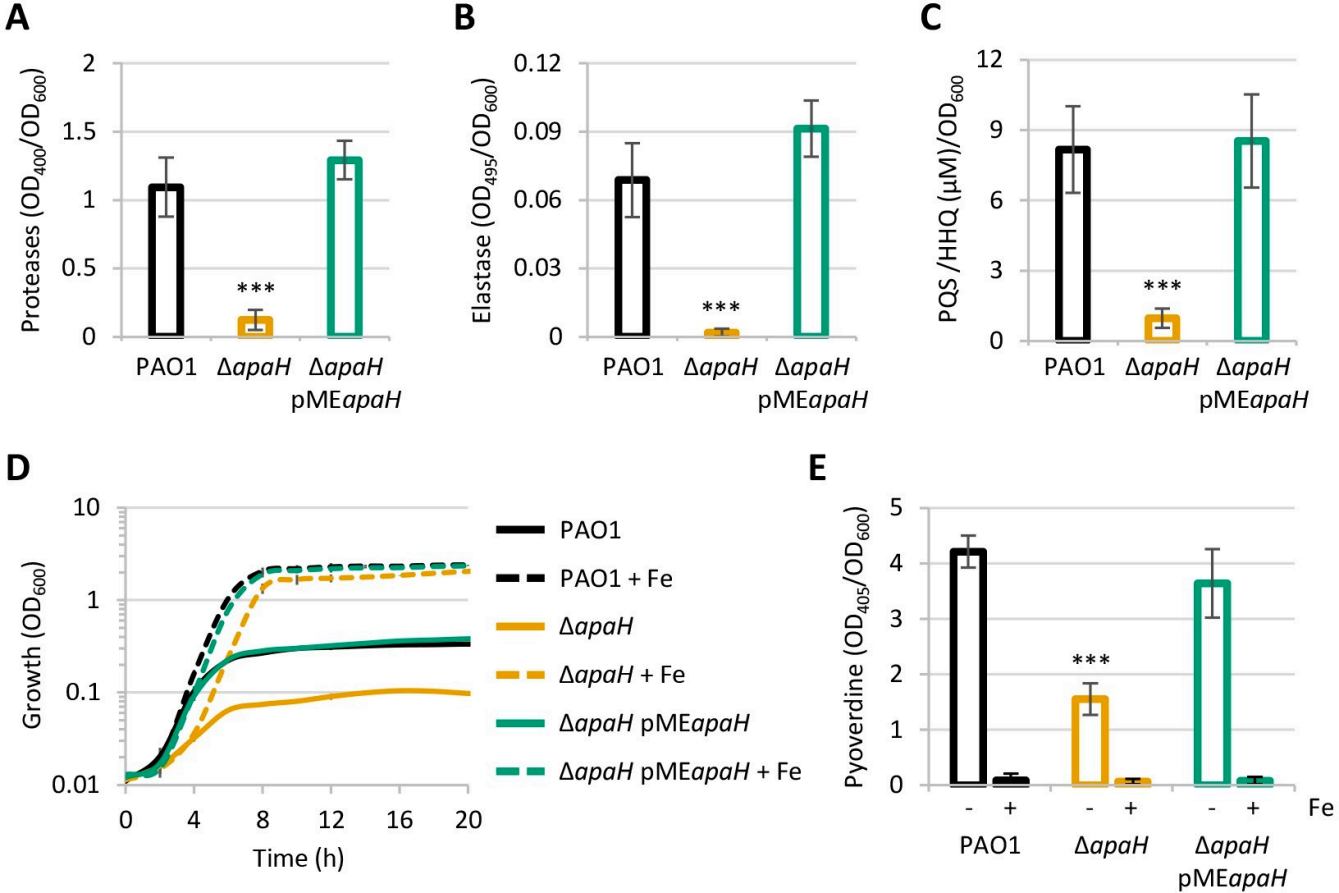
(**A**) Protease activity, (**B**) elastase activity, and (**C**) PQS/HHQ levels, normalized to cell density (OD_600_), in the supernatants of *P*. *aeruginosa* PAO1 and the *apaH* mutant, carrying or not the pME*apaH* plasmid, cultured at 37°C in LB, supplemented with 100 μM IPTG in the case of Δ*apaH* pME*apaH*. (**D**) Growth curves and (**E**) pyoverdine levels, normalized to cell density (OD_600_), of the same strains cultured at 37°C in the iron-poor medium CAA, supplemented with 50 μM FeCl_3_ when indicated (+ Fe), and 100 μM IPTG in the case of Δ*apaH* pME*apaH*. Values are the mean (± standard deviation) of at least three independent assays. Asterisks indicate a statistically significant difference (*P* < 0.001) with respect to PAO1 (ANOVA). The control strains with the empty plasmid pME6032 are shown in S6 Fig.

The RNA-seq analysis highlighted a relevant downregulation of many virulence genes in the *apaH* deletion mutant. We attempted to validate the transcriptomic data by comparing the production of selected virulence factors between the wild type and Δ*apaH* strains. The production of proteases, elastase and *pqs* signal molecules was almost completely abrogated in ApaH-deficient compared to wild type cells (Fig 4A–4C). To confirm that the effect of *apaH* deletion on the *pqs* QS system was specific, we also quantified the QS signal molecules of the *las* and *rhl* QS systems, whose synthase genes (*i*.*e*., *lasI* and *rhlI*) were not identified among DEGs by RNA-seq (S1 Tables). No significant differences were observed in *las* and *rhl* signal production between wild type and Δ*apaH* cells (S3 Fig).

As discussed above, many genes involved in iron metabolism were dysregulated in ApaH-deficient cells. Accordingly, the *apaH* mutant was strongly impaired in growth in an iron-poor minimal medium (CAA), unless an excess of iron was exogenously provided (Fig 4D). This result suggests that the growth defect of the *apaH* mutant in CAA is primarily due to its inability to efficiently acquire and/or use iron. The production of the siderophore pyoverdine was indeed significantly reduced in cells lacking ApaH (Fig 4E), providing further phenotypic validation of RNA-seq data. Interestingly, increasing concentrations of iron promoted growth in a similar way in the Δ*apaH* and parental strains (S4 Fig). This implies that, although probably defective in iron acquisition, ApaH-deficient cells are still able to sense and utilize iron for cell metabolism. Notably, since the QS signal molecule PQS can bind iron and influence iron uptake (reviewed in 34), it cannot be excluded that the reduced production of PQS could at least in part account for the dysregulated iron metabolism observed in *P*. *aeruginosa* cells lacking ApaH.

RNA-seq analysis also revealed that many genes encoding chaperones and other heat shock proteins are downregulated in ApaH-deficient *P*. *aeruginosa* cells (S1 Table and Fig 3). Accordingly, we found that these cells are more sensitive to heat shock than wild type cells (S5 Fig), as previously observed in *E*. *coli* [25]. In contrast, while Ap4A accumulation was reported to increase susceptibility to oxidative stress in some bacteria [25,29], we did not observe dysregulation of the main oxidative stress response genes (S1 Table) or decrease in resistance to hydrogen peroxide or the superoxide-generating agent paraquat in the *P*. *aeruginosa apaH* mutant (S2 Table), further confirming that the effects of Ap4A may vary among different bacterial species.

As expected, the production of virulence factors and *pqs* signal molecules, as well as the ability to thrive under iron-depleted conditions and to tolerate heat shock, was restored to wild type levels by ectopic expression of *apaH* in Δ*apaH* cells (Figs 4, S5 and S6), confirming that the defective phenotypes were due to ApaH deficiency and/or Ap4A intracellular accumulation.

### ApaH inactivation reduces *P*. *aeruginosa* pathogenicity in different infection models

Since ApaH is required for full expression of several *P*. *aeruginosa* virulence genes *in vitro*, this enzyme could represent a novel target to reduce *P*. *aeruginosa* pathogenicity. To test this hypothesis, we assessed the pathogenicity of ApaH-deficient cells in well-established plant and animal models, including lettuce leaves [35], *Galleria mellonella* insect larvae [36], and a mouse model of pulmonary infection [37].

**Fig 5:**
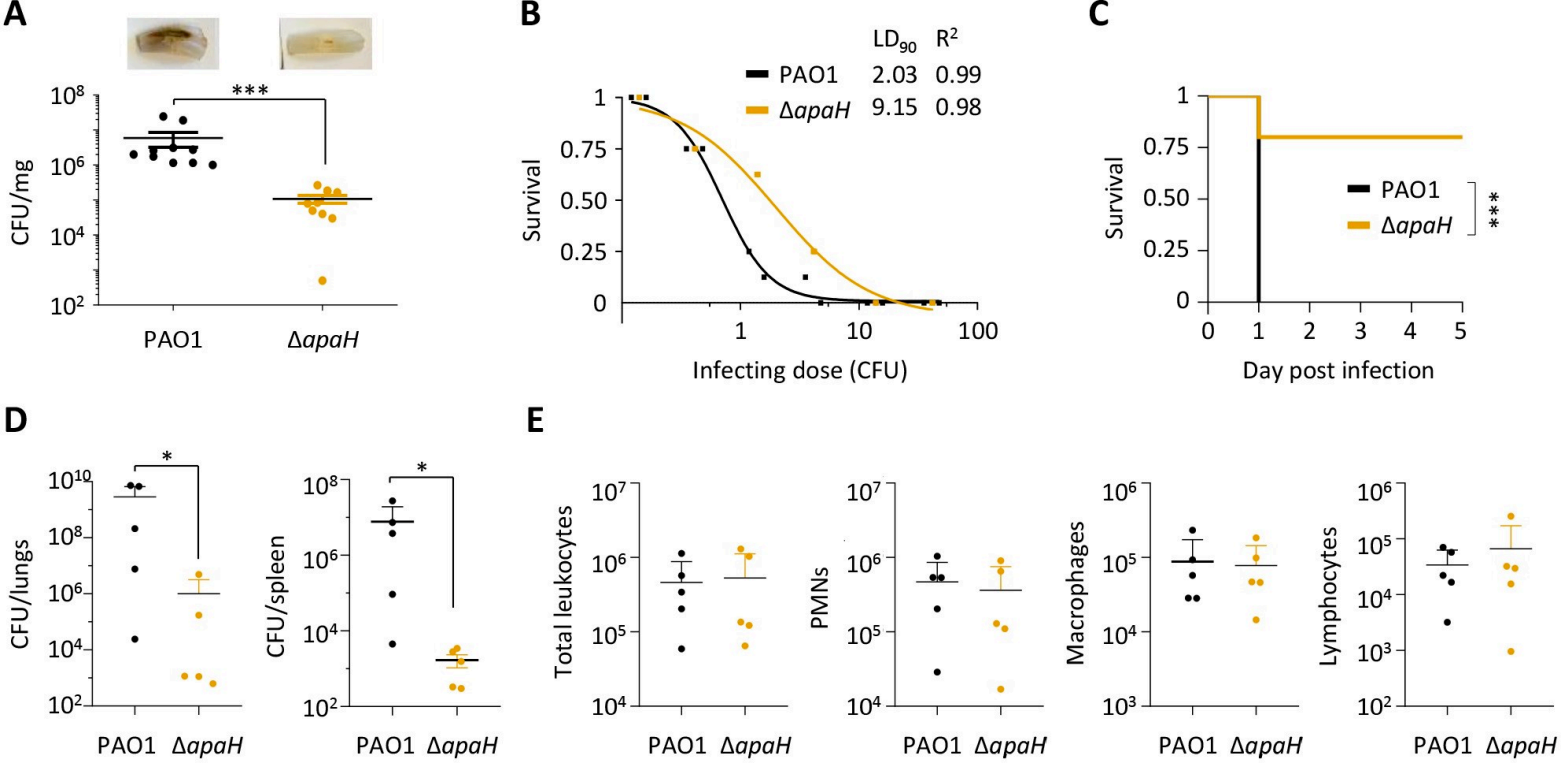
(**A**) Pathogenicity of *P*. *aeruginosa* PAO1 and the *apaH* mutant in the lettuce leaf virulence assay. Bacterial viable cells (CFUs) per mg of lettuce midribs at three days post injection are shown. Two independent experiments were performed, each with five biological replicates. A representative picture of the infected midribs is shown for each strain. (**B**) Dose-dependent survival curves of *G*. *mellonella* larvae infected with different doses of PAO1 or Δ*apaH* cells. Lethal dose 90% (LD_90_) and R^2^ values are shown in the figure. (**C)** Survival of mice (n = 5 per group) challenged with intratracheal injection of 10^7^ CFUs of PAO1 or Δ*apaH*. **(D)** Total bacterial CFU in lungs or spleen from mice (n = 5 per group) intra-tracheally infected with 10^7^ CFU of PAO1 or Δ*apaH* and sacrificed within 24 h of infection (see Materials and methods for details). **(E)** Count of total leukocytes, polymorphonuclear neutrophils (PMN), macrophages, and lymphocytes in BALF from the mice described in panel D. Asterisks indicate statistically significant differences (*P < 0.05, *** *P* < 0.001) with respect to PAO1 (Mann-Whitney test for data in panels A, D, and E; Mantel-Cox test for data in panel C).

In the lettuce leaf infection assay, the wild type strain caused severe rot (black coloration) of the entire midrib, while the Δ*apaH* strain induced some signs of tissue damage only at the inoculation site. Moreover, determination of colony forming units (CFUs) in infected midribs showed a 2-log reduction of bacterial load for the *apaH* mutant compared to the wild type strain (Fig 5A), indicative of impaired growth and/or persistence of ApaH-deficient cells *in planta*.

The *apaH* mutant also showed reduced infectivity in *G*. *mellonella* larvae, with a lethal dose 90% (LD_90_) almost 5-fold higher than that of the wild type strain (Fig 5B). Accordingly, when the same (lethal) dose was injected, lethality was significantly delayed in larvae infected with ApaH-deficient cells with respect to those infected with wild type cells (S7 Fig).

Finally, the Δ*apaH* mutant was much less virulent than the wild type in a mouse model of acute lung infection, as 80% of mice infected with the mutant survived and fully recovered, while 100% of those infected with the wild type strain died (Fig 5C). This result prompted us to investigate the capability of Δ*apaH* mutant cells to persist in and spread from mice lungs. To this aim, mice were intra-tracheally infected and sacrificed 24 h post infection. Wild type infected mice showed high bacterial loads in the lungs and also in the spleen, indicative of efficient bacterial dissemination in the bloodstream. In contrast, mice infected with the *apaH* mutant showed lower persistence in the lungs and poor systemic dissemination (Fig 5D). No differences were observed in the number of leukocytes in bronchoalveolar lavage fluids between wild type- and Δ*apaH*-infected mice, including neutrophils (that are first responder immune cells following acute infections), macrophages (that have key roles in pathogen recognition), and total lymphocytes (that are mediators of cellular adaptive immunity during infections) (Fig 5E). This suggests that the immune system efficiently recognizes ApaH-deficient cells and that wild type cells better escape the host inflammatory response, reaching higher cell densities in the lungs and better disseminating in secondary organs like the spleen.

In conclusion, these *in vivo* assays demonstrate that the lack of ApaH severely impairs *P*. *aeruginosa* PAO1 persistence and infectivity in various infection models.

To further evaluate the impact of Ap4A levels and ApaH activity on virulence, we wondered whether ApaH overexpression and the consequent reduction in Ap4A levels (S1 Fig) could affect the virulence of the wild type strain PAO1. In line with the results of the phenotypic assays (S6 Fig), ApaH overexpression resulted in a minor increase in the mRNA levels of some virulence genes previously identified as DEGs by RNA-seq, and did not affect infectivity in lettuce leaves (S8 Fig). In contrast, ApaH-overexpressing PAO1 cells appeared more virulent than the corresponding empty-plasmid controls in the *G*. *mellonella* infection model (S8 Fig). While these results show an inverse correlation between Ap4A intracellular levels and *P*. *aeruginosa* virulence, they also demonstrate that the negative effect caused by Ap4A accumulation on virulence is much more pronounced than the positive effect caused by Ap4A reduction. This highlights the importance of *P*. *aeruginosa* ApaH in maintaining the intracellular concentration of Ap4A below levels that would be detrimental to virulence gene expression, thus corroborating ApaH as a potential target for antivirulence therapy against *P*. *aeruginosa*.

### The impact of ApaH on virulence is conserved in *P*. *aeruginosa* clinical isolates

*P*. *aeruginosa* strains are extremely diverse at the genetic level [38], and versatility across strains was also observed in the regulatory networks that control metabolism, stress response, and virulence [39–41]. Therefore, we decided to verify whether the ApaH-mediated control of virulence traits is conserved in *P*. *aeruginosa* strains other than PAO1.

**Fig 6:**
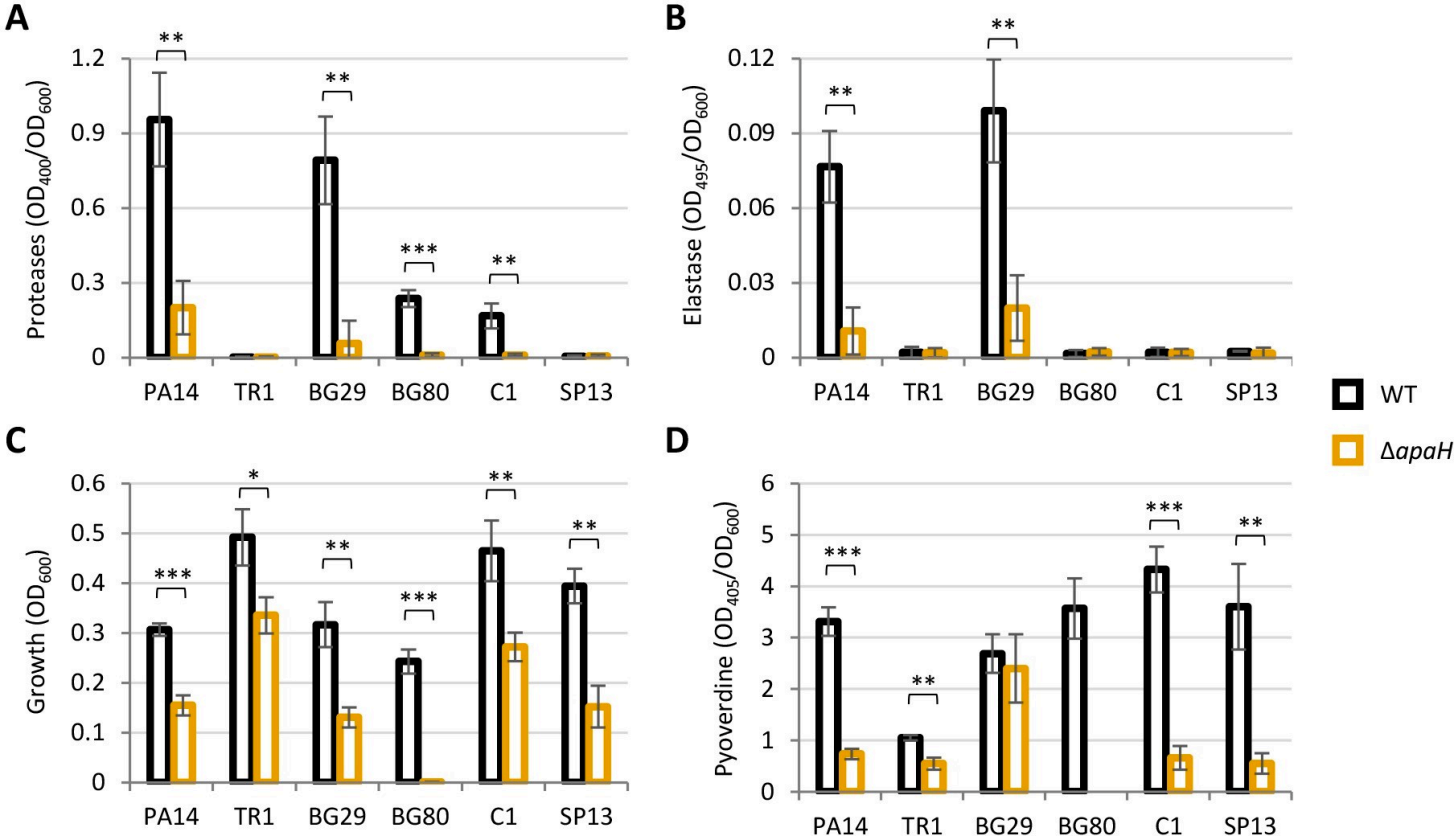
(**A**) Protease and (**B**) elastase activity, normalized to cell density (OD_600_), in the supernatants of *P*. *aeruginosa* PA14 and the indicated *P*. *aeruginosa* clinical isolates (WT) or the cognate *apaH* deletion mutants (Δ*apaH*) cultured at 37°C in LB. (**C**) Maximum growth yields and (**D**) pyoverdine levels normalized to cell density (OD_600_), of the same strains cultured at 37°C in the iron-poor medium CAA. Pyoverdine levels were not measured for BG80 Δ*apaH* as this mutant did not grow in CAA (see panel C). Values are the mean (± standard deviation) of three independent assays. Asterisks indicate statistically significant differences between each Δ*apaH* mutant and the corresponding parental strain (* *P* < 0.05, ** *P* < 0.01, *** *P* < 0.001; unpaired *t* test).

To this aim, we first generated an *apaH* deletion mutant in *P*. *aeruginosa* PA14, another widely used reference strain that is distantly related to PAO1 [42,43]. Phenotypic assays confirmed that ApaH is important for virulence gene expression and adaptation to iron deficiency also in PA14. Indeed, the PA14 *apaH* mutant mirrored the behavior of the PAO1 *apaH* mutant, being significantly impaired in protease, elastase and siderophore production, as well as in the ability to grow under iron-depleted conditions (Fig 6).

Then, we attempted to generate a small collection of *apaH* deletion mutants from *P*. *aeruginosa* clinical isolates. Although we started with ten isolates, we obtained *apaH* mutants only for five of them (S3 Table), including strains isolated from CF lungs (*i*.*e*., TR1, BG29, and BG80) [44,45] and strains from bloodstream infections (*i*.*e*., C1 and SP13) [46,47]. Notably, the remaining isolates were not genetically intractable, as transconjugants with the suicide plasmid integrated into the chromosome were obtained for all clinical strains. Thus, the difficulty in deleting *apaH* suggests that, in some *P*. *aeruginosa* strains, the intracellular accumulation of Ap4A might be highly detrimental to cell growth and/or viability. However, this hypothesis has not been experimentally investigated in this study. Expression of virulence factors was highly variable among clinical isolates, as two and four of them produced almost undetectable levels of proteases and elastase, respectively, under the conditions tested (Fig 6A and 6B). Nonetheless, *apaH* inactivation significantly reduced protease and elastase production in the isolates that released quantifiable amounts of these virulence factors (Fig 6A and 6B). Moreover, growth in an iron-depleted medium was significantly impaired for all *apaH* mutants, although at different extents (Fig 6C). This growth defect could be almost completely abolished by exogenous iron supplementation (S9 Fig). Finally, all *apaH* mutants but BG29Δ*apaH* showed lower pyoverdine production with respect to the corresponding parental strain (Fig 6D).

**Fig 7:**
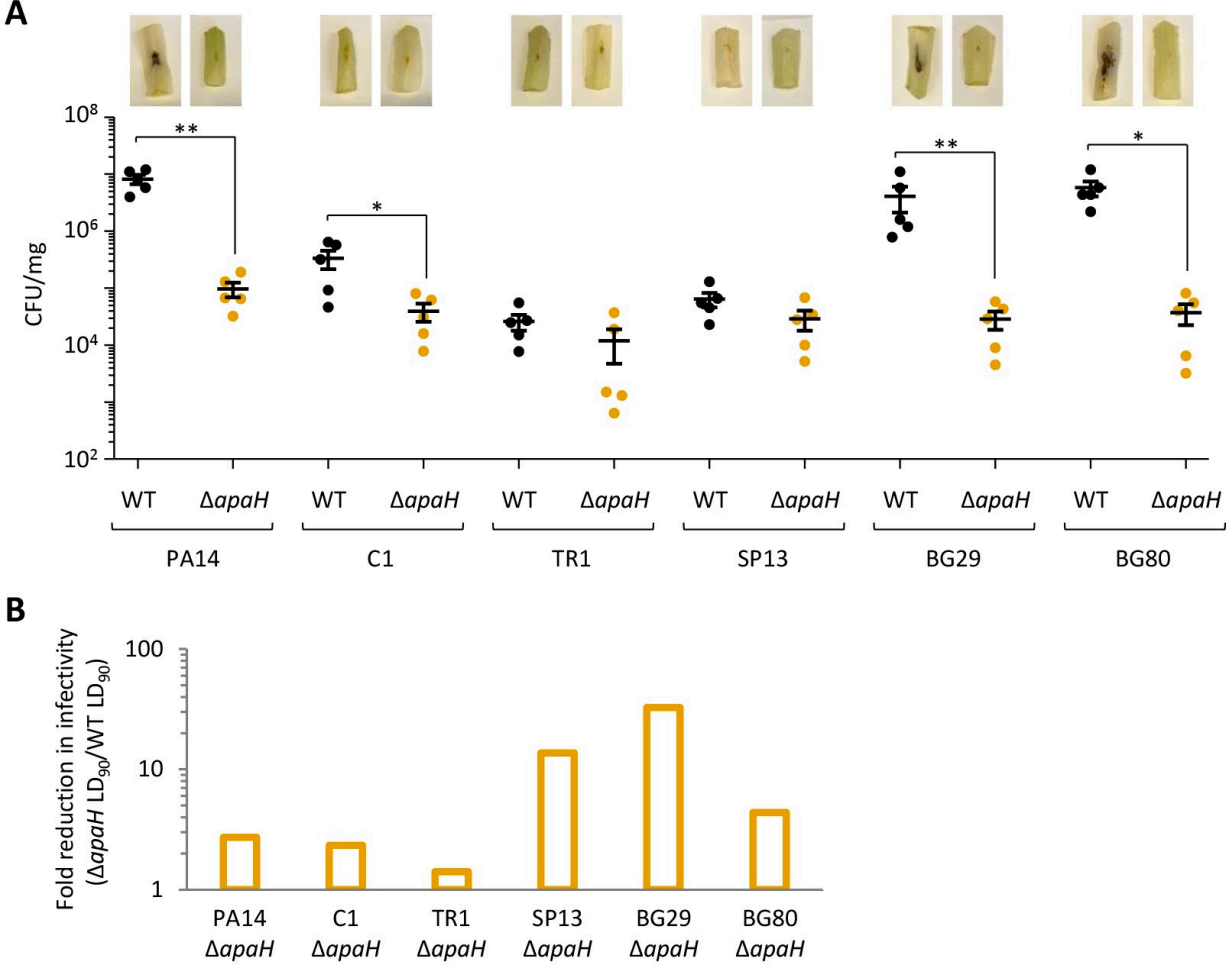
(**A**) Pathogenicity of *P*. *aeruginosa* PA14 and the indicated *P*. *aeruginosa* clinical isolates (WT) or the cognate *apaH* deletion mutants (Δa*paH*) in the lettuce leaf virulence assay. Bacterial viable cells (CFUs) per mg of lettuce midribs at three days post injection are shown. Five biological replicates for each strain were analyzed. A representative picture of the infected midribs is shown for each strain. (**B**) Reduction in infectivity, calculated as the ratio between the lethal dose 90% (LD_90_) of the Δa*paH* mutant and the LD_90_ of the corresponding wild type strain, observed for *apaH* mutants of *P*. *aeruginosa* PA14 and the indicated *P*. *aeruginosa* clinical isolates in *G*. *mellonella* larvae. The dose-dependent survival curves used to calculate the LD_90_ values and the corresponding R^2^ values are shown in S10 Fig.

Finally, we used the lettuce and *G*. *mellonella* infection models to confirm that ApaH is important for pathogenicity also in PA14 and clinical isolates. As shown in Fig 7A, *apaH* deletion significantly impaired infectivity in lettuce leaves in all but two isolates. Interestingly, these two isolates (TR1 and SP13) showed poor ability to proliferate and cause damage in lettuce leaves, at levels comparable to the Δ*apaH* mutants of the other strains (Fig 7A), suggesting that the modest effect of ApaH may be due to poor virulence of these two strains in the lettuce model of infection. All the Δ*apaH* mutants also resulted less virulent than the parental strains in *G*. *mellonella* larvae, although great variability was observed among isolates, as the increase in the LD_90_ values with respect to the corresponding parental strains ranged between 1.4 fold for TR1 Δ*apaH* and 30 fold for BG29 Δ*apaH* (Figs 7B and S10).

Although the number of clinical isolates analyzed in this study is small, overall these analyses indicate that the positive effect of ApaH on *P*. *aeruginosa* virulence is not restricted to laboratory-adapted strains.

## Conclusions

Although first discovered more than 50 years ago, the physiological role of Ap4A remains elusive [20]. The role of Ap4A in bacteria has been studied mainly by generating mutant strains defective in Ap4A-degrading enzyme(s), which accumulate Ap4A at high intracellular levels. This approach highlighted pleiotropic and variable effects caused by Ap4A accumulation in different bacteria, including loss of motility, higher heat and oxidative stress sensitivity, lower antibiotic tolerance, reduced invasiveness in mammalian cells, and/or enhanced biofilm-forming capability [24–30]. Here, we showed that the lack of the diadenosine tetraphosphatase ApaH strongly reduces the infectivity of the difficult-to-treat pathogen *P*. *aeruginosa* in different infection models, in line with the downregulation of many virulence genes observed *in vitro*. In contrast, no relevant effect of ApaH was observed on biofilm formation, oxidative stress and antibiotic resistance in *P*. *aeruginosa*. We also provided evidence that the importance of ApaH for virulence gene expression may be conserved in clinical isolates, although our analysis was limited to a small number of strains.

The mechanisms by which Ap4A exert its effects in bacterial cells are still elusive. Proteomic studies with Ap4A-derived probes identified several putative Ap4A targets in *E*. *coli*, including chaperones, stress response proteins, and core metabolic enzymes [25,48,49]. Recently, the interaction of Ap4A with inosine-5′-monophosphate dehydrogenase (IMPDH), a key enzyme for nucleotide biosynthesis, has been thoroughly characterized in *Bacillus subtilis* [50]. Thus, it is plausible that Ap4A can regulate at least some phenotypes by binding to specific protein partners. On the other hand, it has been recently demonstrated that Ap4A can be incorporated by *E*. *coli* RNA polymerase into nascent transcripts as a protective 5’-cap that increases RNA stability [51,52], and that specific promoter features can affect the efficiency of RNA capping [53]. This finding raises the possibility that the phenotypes observed for mutants lacking the ApaH enzyme might not only result from Ap4A-mediated control of protein effectors, but also from altered RNA lifetimes and consequent modulation of gene expression. The evidence that ApaH plays an important role in *P*. *aeruginosa* virulence gene regulation will hopefully drive future biochemical and genetic studies to investigate the molecular mechanisms underlying ApaH-mediated control of gene expression in this bacterium.

The spread of drug resistance in bacterial pathogens has pushed the researchers toward searching for antibiotic alternatives, including compounds with antivirulence activity [54,55]. This work provides the first direct evidence that ApaH inactivation could be a suitable strategy to inhibit bacterial pathogenicity *in vivo*. Notably, phosphatases distantly related to bacterial ApaH enzymes, termed ApaH-like phosphatases (ALPHs), have been identified in some eukaryotes but not in Vertebrata [56,57], implying that specific ApaH inhibitors should not affect Ap4A homeostasis in human cells. Early studies developed and tested substrate analogue inhibitors of *E*. *coli* ApaH, mainly to characterize the biochemical properties of the enzyme [58]. These studies, combined with the structural-functional characterization of *P*. *aeruginosa* ApaH, could pave the way for the design of ApaH inhibitors to be tested and developed as antivirulence drugs to treat *P*. *aeruginosa* infections.

## Materials and methods

### Ethics statement

Animal protocol was approved by the Italian Ministry of Health (Approval # 855) according to the Italian legislative decree 26/2014.

### Bacterial strains, plasmids, and growth conditions

The strains and plasmids used in this study are listed in S3 Table. Bacteria were routinely cultured in LB (Lennox formulation) for genetic manipulation. For specific assays, LB, Mueller-Hinton (MH), Tryptic Soy Broth (TSB), or Casamino acids (CAA) medium (0.5% Casamino acids, 0.4 mM MgCl_2_) [59] were used, as indicated in the text. When required, antibiotics were added at the following concentration for *E*. *coli* (the concentrations used for *P*. *aeruginosa* are shown in brackets): ampicillin, 100 μg/mL; tetracycline, 12.5 μg/mL (50–100 μg/mL); carbenicillin (500 μg/mL); nalidixic acid, 15 μg/mL; chloramphenicol, 30 μg/mL (375–650 μg/mL). When specified, growth media were supplemented with isopropyl β-d-1-thiogalactopyranoside (IPTG) or FeCl_3_ at the indicated concentrations.

### Generation of mutants

The *apaH* deletion mutants were generated using the previously described suicide plasmid pDM4Δ*apaH*, which contains the upstream and downstream regions of the *apaH* coding sequence cloned adjacent to each other [31]. The plasmid pDM4Δ*apaH* was transferred into *P*. *aeruginosa* strains by conjugation, and transconjugants were selected on LB agar plates containing 15 μg/mL nalidixic acid and 375 μg/mL chloramphenicol. For some clinical strains, chloramphenicol concentration was increased up to 650 μg/mL. Deletion mutants were obtained by homologous recombination and sucrose-based selection as previously described [60], screened by colony PCR using the primer pair apaH↑_FW/apaH↓_RV [31], and confirmed by DNA sequencing.

### Growth assays

Planktonic growth assays were performed either in flasks or in microtiter plates, as indicated. Bacterial strains were precultured in LB and then refreshed 1:1,000 in LB or CAA. Bacterial cultures were incubated in flasks at 37°C and 200 rpm or aliquoted in the wells of 96-well microtiter plates (200 μL in each well) and incubated at 37°C in a Spark 10M microtiter plate reader (Tecan). Growth was measured over time as the optical density at 600 nm (OD_600_) of the bacterial cultures.

### Biofilm assay

Biofilm formation was assessed through the microtiter dish biofilm assay [61], with minor modifications. Briefly, bacterial cells cultured overnight in LB were inoculated in the same medium at OD_600_ = 0.002 and aliquoted in 96-well polystyrene microtiter plates (200 μL per well). Five replicate wells for each strain were used in each experiment. After 24-h incubation at 37°C under static conditions, the OD_600_ of the bacterial cultures was measured in a Spark 10M microtiter plate reader (Tecan). Then, planktonic cells were removed, wells were washed four times with distilled water, and attached cells were stained with 0.1% crystal violet for 15 min. After four washes with water, biofilm-bound dye was eluted with 30% acetic acid for 15 min and the OD_550_ was measured in a Spark 10M microtiter plate reader (Tecan). For each well, the OD_550_ value was divided by the OD_600_ of the bacterial culture to normalize biofilm formation to planktonic growth.

### MIC assays

Antibiotic, H_2_O_2_ and paraquat MICs were determined through the broth microdilution method. Strains were precultured in MH and then refreshed at ca. 5×10^5^ cells/mL in the same medium supplemented with increasing concentrations of each antibiotic/compound in 96-well microtiter plates. MIC was visually recorded after 24-h incubation at 37°C. At least three independent experiments were performed for each strain/antibiotic.

### Kirby-Bauer disc diffusion assay

Bacterial cell suspensions in saline were normalized at 0.5 McFarland Standard and swabbed onto MH agar plates. Discs containing gentamicin (10 μg), streptomycin (10 μg), tobramycin (10 μg), ciprofloxacin (5 μg), erythromycin (15 μg), novobiocin (30 μg), rifampicin (5 μg), or imipenem (10 μg) (Becton Dickinson) were placed on the surface of the inoculated plates, and growth inhibition halo diameters were measured after 24-h incubation at 37°C.

### Time-kill assays

Late-exponential phase bacterial cultures in MH were diluted in the same medium at ca. 5×10^5^ CFU/mL in the presence of gentamicin or kanamycin concentrations corresponding to 1× or 2×MIC. Bacterial cultures were incubated at 37°C with vigorous shaking (200 rpm) and, at different time points, serial dilutions were prepared in saline and plated on MH agar plates for CFU counting.

### Quantification of intracellular nucleotide levels

Cells for Ap4A, c-di-GMP, ATP, ADP, GTP, and GDP quantification were collected after 12 h of growth in LB at 37°C. Both extraction and quantification of Ap4A and c-di-GMP by liquid chromatography coupled with tandem mass spectrometry were performed as previously described [62,63]. ATP, ADP, GTP and GDP were extracted with the same protocol used for Ap4A and quantified under the same chromatographic conditions [62]. Mass spectrometric detection was performed in positive ionization mode, with mass transitions as follows: ATP m/z: 508 → 136; ADP m/z: 428 → 136; GTP m/z: 524 → 152; GDP 444 → 152. The amounts of Ap4A, c-di-GMP, ATP, ADP, GTP, and GDP were normalized to the cellular protein content of the corresponding cell extracts determined using the DC protein assay kit (Bio-Rad) and bovine serum albumin as the standard.

### RNA extraction, RNA-seq and RT-qPCR

Total RNA was extracted from three independent biological replicates for each sample. Bacteria were cultured in LB at 37°C until mid-exponential growth phase. One-mL aliquots of bacterial cultures were mixed with 2 mL of RNA Protect Bacteria Reagent (Qiagen), and RNA was purified using RNeasy Mini Kit (Qiagen). Eluted RNA samples were treated with TURBO DNase (Thermo Fisher Scientific) and SUPERase-In (Ambion) for 1 h at 37°C. DNase I was removed upon RNA purification with the RNeasy Column Purification Kit (Qiagen). RNA-seq was performed at GENEWIZ (Azenta Life Sciences, Leipzig, Germany). RNA quantification and quality assessment, rRNA depletion, library preparation, sequencing, and statistical analysis of the data were performed as previously described [64]. The Wald test was used to generate *P* values and log2 fold changes that were converted to FCs. Only DEGs with an adjusted *P* value < 0.05 were considered statistically significant (S1 Table). RNA-seq data have been deposited in the NCBI Gene Expression Omnibus database [65] and are accessible through GEO Series accession number GSE255863 (https://www.ncbi.nlm.nih.gov/geo/query/acc.cgi?acc=GSE255863).

For quantitative reverse transcription PCR (RT-qPCR), cDNA was reverse transcribed from 0.5 μg of total RNA with Prime Script RT Reagent Kit (Takara). The cDNA was used as the template for RT-qPCR in a AriaMx Real-Time PCR System (Agilent) using TB Green Premier EX Taq master mix (Takara). The primers used for RT-qPCR are listed in S4 Table. Relative expression of each gene with respect to the housekeeping gene *rpoD* was calculated using the 2^-ΔΔCt^ method [66].

### Virulence factor assays and quantification of QS signal molecules

Total protease and LasB activities were measured in cell-free supernatants, obtained from bacterial cultures grown for 8 h in LB, using the azocasein and elastin-Congo red assays, respectively, as previously described [67].

Pyoverdine was measured as the optical density at 405 nm (OD_405_) of cell-free supernatants obtained from bacterial cultures grown for 20 h in CAA, appropriately diluted in 100 mM Tris-HCl (pH 8.0), and then normalized to the OD_600_ of the corresponding bacterial culture [45].

Levels of 3OC_12_-HSL, C_4_-HSL, and PQS/HHQ signal molecules were determined in cell-free supernatants, obtained from bacterial cultures grown for 8 h in LB, using the biosensor strains PA14-R3 [68], C4-HSL-Rep [69], and AQ-Rep [70] (S3 Table), as previously described [64], and then normalized to the OD_600_ of the corresponding bacterial culture.

### Heat shock sensitivity assay

Heat sensitivity of *P*. *aeruginosa* cells was assessed as previously described [71]. Briefly, cells were cultured in LB at 20°C until late-exponential growth phase, harvested by centrifugation, resuspended in saline, and then incubated at 50°C. After 30, 60, 120 and 240 min of incubation, aliquots of the cell suspensions were collected, serially diluted in saline and plated on LB agar plates for CFU counting.

### Infection assays

The lettuce leaf infection assay was performed as described [34], with few modifications. Briefly, 10 μL of bacterial cells resuspended at an OD_600_ of 1 in 10 mM MgSO_4_ were inoculated into the midribs of fresh Romaine lettuce leaves. The midribs were incubated for three days at 30°C, photographed, and ground in saline with a plastic pestle until a nearly homogenous mixture was obtained. Serial ten-fold dilutions were prepared in saline and plated onto LB agar plates for viable cell counting.

The *G*. *mellonella* infection assay was performed by injecting serial dilutions of bacterial cell suspensions in saline into the hemolymph of *G*. *mellonella* larvae as described [35]. Eight larvae were infected with each infecting dose and two independent experiments were performed. Infected larvae were incubated at 30°C for up to three days to monitor mortality. Kaplan-Meier curves, dose-response curves, LD_90_ and R^2^ values were determined using GraphPad Prism as previously described [72].

For mouse infection assays, 10^7^ mid-exponential cells of PAO1 or Δ*apaH*, cultured in TSB and resuspended in Dulbecco’s phosphate buffered saline (DBPS), were intra-tracheally injected into C57Bl6/N mice (8–12 week-old, 20–22 g, Charles River), previously anesthetized with an intraperitoneal injection of 500 mg/kg of 2,2,2,-tribromoethanol. For assessing lethality, mice were monitored daily and sacrificed with CO_2_ inhalation when they reached a humane endpoint with weight loss > 20% and evidence of severe clinical disease (*e*.*g*., scruffy coat, loss of appetite, severe inactivity, or painful posture). Determination of bacterial load in lungs and spleen and analysis of leukocytes in bronchoalveolar lavage fluids (BALF) were carried out as previously reported [36] for infected mice sacrificed with CO_2_ inhalation at 24 h post infection.

### Statistical analysis

Statistical analyses were performed with the software GraphPad Instat, using the unpaired *t* test, ANOVA test, Mann-Whitney test or Mantel-Cox test, as indicated in the figure legends.

## Supporting information

S1 Table DEGs between PAO1 and Δ*apaH* cells identified by RNA-seq. (XLSX)

S2 Table MIC of hydrogen peroxide (H_2_O_2_) and paraquat (PQ) for *P*. *aeruginosa* PAO1 and the isogenic Δ*apaH* mutant. (PDF)

S3 Table Bacterial strains and plasmids used in this study. (PDF)

S4 Table Primers used for RT-qPCR. (PDF)

S1 Fig (**A**) Growth curves of the wild type strain *P*. *aeruginosa* PAO1 and the *apaH* mutant carrying the pME*apaH* plasmid or the empty plasmid pME6032, cultured at 37°C in LB supplemented with 100 μM IPTG. (**B**) Intracellular Ap4A levels of the same strains cultured for 12 h under the conditions described in panel A. Values are the mean (± standard deviation) of three independent experiments. Asterisks indicate a statistically significant difference (*P* < 0.01) with respect to PAO1 pME6032 (ANOVA).
(PDF)

S2 Fig (**A**) Intracellular levels of ATP, ADP, GDP and GTP in *P*. *aeruginosa* PAO1 and the *apaH* mutant cultured at 37°C in LB. (**B**) Intracellular levels of ATP, ADP, GDP and GTP in *P*. *aeruginosa* PAO1 and the *apaH* mutant carrying the empty plasmid pME6032 or the plasmid pME*apaH*, cultured at 37°C in LB supplemented with 100 μM IPTG. Values are the mean (± standard deviation) of three biological replicates. Asterisks indicate a statistically significant difference (*P* < 0.05) with respect to PAO1 (panel A; unpaired *t* test) or PAO1 pME6032 (panel B; ANOVA).
(PDF)

S3 Fig Levels of the *rhl* QS signal molecule C_4_-HSL (**A**) and the *las* QS signal molecule 3OC_12_-HSL (**B**), normalized to cell density (OD_600_), in the supernatants of *P*. *aeruginosa* PAO1 and the *apaH* mutant, carrying or not the empty plasmid pME6032 or the plasmid pME*apaH*, cultured at 37°C in LB, supplemented with 100 μM IPTG in the case of strains carrying the plasmids. Values are the mean (± standard deviation) of three independent assays. No statistically significant differences (*P* > 0.05) were observed with respect to PAO1 unpaired *t* test) or PAO1 pME6032 (ANOVA).
(PDF)

S4 Fig Growth curves of *P*. *aeruginosa* PAO1 and the *apaH* mutant in the iron-poor medium CAA supplemented or not with the indicated concentrations of FeCl_3_ in microtiter plates at 37°C in an automatic microtiter plate reader. Values are the mean of three technical replicates and the curves are representative of three biological replicates.
(PDF)

S5 Fig Heat tolerance of (**A**) *P*. *aeruginosa* PAO1 and the *apaH* mutant or (**B**) the same strains carrying the empty plasmid pME6032 or the plasmid pME*apaH*. Cells were cultured at 20°C in LB, supplemented with 100 μM IPTG for plasmid-harboring strains, and subjected to a heat shock at 50°C. Cell viability was monitored as CFU/mL at different time points. Values are the mean (± standard deviation) of at least three independent assays. Asterisks indicate a statistically significant difference (*P* < 0.05) with respect to PAO1 (panel A; unpaired *t* test) or PAO1 pME6032 (panel B; ANOVA).
(PDF)

S6 Fig (**A**) Protease activity, (**B**) elastase activity, and (**C**) PQS/HHQ levels, normalized to cell density (OD_600_), in the supernatants of *P*. *aeruginosa* PAO1 and the *apaH* mutant, carrying the empty plasmid pME6032 or the plasmid pME*apaH*, cultured at 37°C in LB supplemented with 100 μM IPTG. (**D**) Growth curves and (**E**) pyoverdine levels, normalized to cell density (OD_600_), of the same strains cultured at 37°C in the iron-poor medium CAA, supplemented with 100 μM IPTG and 50 μM FeCl_3_ when indicated (+ Fe). Values are the mean (± standard deviation) of three independent assays. Asterisks indicate a statistically significant difference (*P* < 0.001) with respect to PAO1 pME6032 (ANOVA).
(PDF)

S7 Fig Kaplan-Meier survival curves of *G*. *mellonella* larvae infected with 13.8 (± 2.8) PAO1 cells or 13.9 (± 0.2) Δ*apaH* cells. Sixteen larvae were infected with each strain in two independent experiments. Asterisks indicate a statistically significant difference (*P* < 0.001) with respect to PAO1 (Mantel-Cox test).
(PDF)

S8 Fig (**A**) Relative mRNA levels of selected virulence genes (*prpL*, *pvdD*, *aprA*, *lasA*, *lasB*), determined by RT-qPCR, in *P*. *aeruginosa* PAO1 carrying the empty plasmid pME6032 or the plasmid pME*apaH*, cultured in LB supplemented with 100 μM IPTG until mid-exponential phase. Values are the mean (± standard deviation) of three biological replicates. (**B**) Pathogenicity in the lettuce leaf virulence assay of PAO1 pME6032 and PAO1 pME*apaH* precultured in LB supplemented with 100 μM IPTG. Bacterial viable cells (CFUs) per mg of lettuce midribs at three days post injection are shown. Five biological replicates for each strain were analyzed. Representative pictures of the infected midribs are shown. (**C**) Dose-dependent survival curves of *G*. *mellonella* larvae infected with different doses of PAO1 pME6032 or PAO1 pME*apaH* precultured in LB supplemented with 100 μM IPTG. Lethal dose 90% (LD_90_) and R^2^ values are shown in the figure.
(PDF)

S9 Fig Maximum growth yields of *P*. *aeruginosa* PA14 and the indicated *P*. *aeruginosa* clinical isolates (WT) or the cognate *apaH* mutants (Δ*apaH*) in CAA medium supplemented with 50 μM FeCl_3_ over 24 h of growth at 37°C. Values are the mean (± standard deviation) of three independent assays. The asterisk indicates a statistically significant difference (*P* < 0.05) between the Δ*apaH* mutant and its parental strain C1 (unpaired *t* test).
(PDF)

S10 Fig Dose-dependent survival curves of *G*. *mellonella* larvae infected with different doses of the indicated wild type strains (black lines and symbols) or the corresponding Δ*apaH* mutants (orange lines and symbols). Lethal dose 90% (LD_90_) and R^2^ values are shown in the figure.
(PDF)

S1 Data Raw data of all experiments. (PDF)
