# Supplementary material for: The diadenosine tetraphosphate hydrolase ApaH contributes to Pseudomonas aeruginosa pathogenicity
Source: PLoS Pathog. 2024 Aug 19;20(8):e1012486. doi: 10.1371/journal.ppat.1012486 (PMC11361744; doi:10.1371/journal.ppat.1012486)
Supplement: S2 Table — (PDF) [file ppat.1012486.s002.pdf]

S2 Table. MIC of hydrogen peroxide (H_2O_2) and paraquat (PQ) for *P. aeruginosa* PAO1 and the isogenic Δ*apaH* mutant.

**Compound**	**MIC (mM)[1]**	
	**PAO1**	**Δ*apaH***
H_2O_2	2	2
PQ	0.5	0.5

[1] Values correspond to the mode of three independent experiments.
