# Supplementary material for: The diadenosine tetraphosphate hydrolase ApaH contributes to Pseudomonas aeruginosa pathogenicity
Source: PLoS Pathog. 2024 Aug 19;20(8):e1012486. doi: 10.1371/journal.ppat.1012486 (PMC11361744; doi:10.1371/journal.ppat.1012486)
Supplement: S3 Table — (PDF) [file ppat.1012486.s003.pdf]

S3 Table. Bacterial strains and plasmids used in this study.

Strain/plasmid	Genotype or relevant characteristics	Source or reference
E. coli		
S17.1λ*pir*	*thi pro hsdR hsdM*$^{+}$ *recA* RP4-2-Tc::Mu-Km::Tn7λ*pir,* Gm^{R}	1
P. aeruginosa		
PAO1 (ATCC15692)	Reference isolate, wild type	American Type Culture Collection
PAO1 Δ*apaH*	PAO1 derivative with an in-frame deletion of the *apaH* coding sequence	This study
PA14	Reference isolate, wild type	2
PA14 Δ*apaH*	PA14 derivative with an in-frame deletion of the *apaH* coding sequence	This study
BG29	CF clinical isolate	3
BG29 Δ*apaH*	BG29 derivative with an in-frame deletion of the *apaH* coding sequence	This study
BG80	CF clinical isolate	3
BG80 Δ*apaH*	BG80 derivative with an in-frame deletion of the *apaH* coding sequence	This study
TR1	CF clinical isolate	4
TR1 Δ*apaH*	TR1 derivative with an in-frame deletion of the *apaH* coding sequence	This study
C1	Blood isolate	5
C1 Δ*apaH*	C1 derivative with an in-frame deletion of the *apaH* coding sequence	This study
SP13	Blood isolate	6
SP13 Δ*apaH*	SP13 derivative with an in-frame deletion of the *apaH* coding sequence	This study
KK27	CF clinical isolate	4
BG27	CF clinical isolate	3
BG66	CF clinical isolate	3
BG73	CF clinical isolate	3
BG75	CF clinical isolate	3
**Plasmid**		
pDM4	Suicide vector used for deletion mutagenesis in *P. aeruginosa*; *sacB, oriR6K*; Cm^{R}	7
pDM4Δ*apaH*	Derivative of pDM4 carrying the DNA regions upstream and downstream of the *apaH* coding sequence, used for the in-frame deletion of *apaH* by homologous recombination	8
pME6032	IPTG-inducible expression vector; *lacI*^{Q}, Tc^{R}	9
pME*apaH*	pME6032 derivative containing the *apaH* coding sequence downstream of the IPTG-inducible promoter	8
pFLP2	Broad-host-range plasmid expressing the Flp recombinase, *sacB*; Ap^{R}/Cb^{R}	10

References

1. Simon R, Priefer U, Pühler A. A Broad Host Range Mobilization System for In Vivo Genetic Engineering: Transposon Mutagenesis in Gram Negative Bacteria. Nat Biotechnol 1983; 1:784-791. doi.org/10.1038/nbt1183-784.
2. Rahme LG, Stevens EJ, Wolfort SF, Shao J, Tompkins RG, Ausubel FM. Common virulence factors for bacterial pathogenicity in plants and animals. Science. 1995 Jun 30;268(5219):1899-902. doi: 10.1126/science.7604262.
3. Imperi F, Fiscarelli EV, Visaggio D, Leoni L, Visca P. Activity and Impact on Resistance Development of Two Antivirulence Fluoropyrimidine Drugs in *Pseudomonas aeruginosa*. Front Cell Infect Microbiol. 2019 Mar 11;9:49. doi: 10.3389/fcimb.2019.00049.
4. Bragonzi A, Paroni M, Nonis A, Cramer N, Montanari S, Rejman J, et al. *Pseudomonas aeruginosa* microevolution during cystic fibrosis lung infection establishes clones with adapted virulence. Am J Respir Crit Care Med. 2009 Jul 15;180(2):138-45. doi: 10.1164/rccm.200812-1943OC.

5. Lanini S, D'Arezzo S, Puro V, Martini L, Imperi F, Piselli P, et al. Molecular epidemiology of a *Pseudomonas aeruginosa* hospital outbreak driven by a contaminated disinfectant-soap dispenser. PLoS One. 2011 Feb 16;6(2):e17064. doi: 10.1371/journal.pone.0017064.
6. Bonchi C, Frangipani E, Imperi F, Visca P. Pyoverdine and proteases affect the response of *Pseudomonas aeruginosa* to gallium in human serum. Antimicrob Agents Chemother. 2015 Sep;59(9):5641-6. doi: 10.1128/AAC.01097-15.
7. Milton DL, O'Toole R, Horstedt P, Wolf-Watz H. Flagellin A is essential for the virulence of *Vibrio anguillarum*. J Bacteriol. 1996 Mar;178(5):1310-9. doi: 10.1128/jb.178.5.1310-1319.1996.
8. Cervoni M, Sposato D, Lo Sciuto A, Imperi F. Regulatory Landscape of the *Pseudomonas aeruginosa* Phosphoethanolamine Transferase Gene *eptA* in the Context of Colistin Resistance. Antibiotics (Basel). 2023 Jan 18;12(2):200. doi: 10.3390/antibiotics12020200.
9. Heeb S, Blumer C, Haas D. Regulatory RNA as mediator in GacA/RsmA-dependent global control of exoproduct formation in *Pseudomonas fluorescens* CHA0. J Bacteriol. 2002 Feb;184(4):1046-56. doi: 10.1128/jb.184.4.1046-1056.2002.
10. Hoang TT, Karkhoff-Schweizer RR, Kutchma AJ, Schweizer HP. A broad-host-range Flp-FRT recombination system for site-specific excision of chromosomally-located DNA sequences: application for isolation of unmarked Pseudomonas aeruginosa mutants. Gene. 1998 May 28;212(1):77-86. doi: 10.1016/s0378-1119(98)00130-9.
