# Supplementary material for: The diadenosine tetraphosphate hydrolase ApaH contributes to Pseudomonas aeruginosa pathogenicity
Source: PLoS Pathog. 2024 Aug 19;20(8):e1012486. doi: 10.1371/journal.ppat.1012486 (PMC11361744; doi:10.1371/journal.ppat.1012486)
Supplement: S4 Table — (PDF) [file ppat.1012486.s004.pdf]

S4 Table. Primers used for RT-qPCR.

Primer name	Sequence (5'-3')	Target gene
*prpL*_RT_FW	GCCGGCAAGGAAATCTTC	*prpL*
*prpL*_RT_RV	CAGGGAGTCGGCGAAATAC	
*pvdD*_RT_FW	GAAAGGAAGGCATTGGCTG	*pvdD*
*pvdD*_RT_RV	GTAGACGCAAGACACTCGGG	
*aprA*_RT_FW	CAATGGCCATCCGTCCTAT	*aprA*
*aprA*_RT_RV	TGCCCAGCGAGTAGATATCG	
*lasA*_RT_FW	GGAGCGGCTACTACAGCATC	*lasA*
*lasA*_RT_RV	CTGGCGCAACTGGTATTCCT	
*lasB*_RT_FW	GCGATCATGGGTGTTTCG	*lasB*
*lasB*_RT_RV	TAGCGGGTGACCTGCTTG	
*rpoD*_RT_FW	GGGCGAAGAAGGAAATGGTC	*rpoD*
*rpoD*_RT_RV	CAGGTGGCGTAGGTGGAGAA	
