# Supplementary material for: The diadenosine tetraphosphate hydrolase ApaH contributes to Pseudomonas aeruginosa pathogenicity
Source: PLoS Pathog. 2024 Aug 19;20(8):e1012486. doi: 10.1371/journal.ppat.1012486 (PMC11361744; doi:10.1371/journal.ppat.1012486)
Supplement: S1 Fig — (A) Growth curves of the wild type strain P. aeruginosa PAO1 and the apaH mutant carrying the pMEapaH plasmid or the empty plasmid pME6032, cultured at 37°C in LB supplemented with 100 μM IPTG. (B) Intracellular Ap4A levels of the same strains cultured for 12 h under the conditions described in panel A. Values are the mean (± standard deviation) of three independent experiments. Asterisks indicate a statistically significant difference (P < 0.01) with respect to PAO1 pME6032 (ANOVA). (PDF) [file ppat.1012486.s005.pdf]

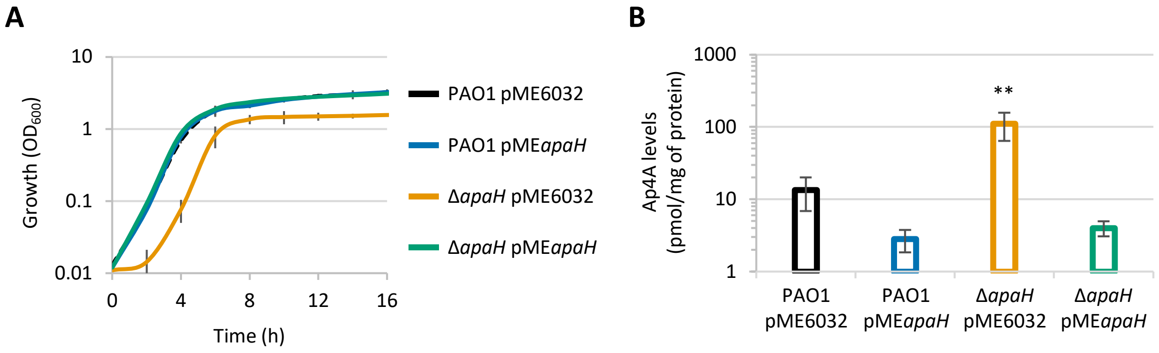

S1 Fig. (**A**) Growth curves of the wild type strain *P. aeruginosa* PAO1 and the *apaH* mutant carrying the pME*apaH* plasmid or the empty plasmid pME6032, cultured at 37°C in LB supplemented with 100 µM IPTG. (**B**) Intracellular Ap4A levels of the same strains cultured for 12 h under the conditions described in panel A. Values are the mean (± standard deviation) of three independent experiments. Asterisks indicate a statistically significant difference ($P < 0.01$) with respect to PAO1 pME6032 (ANOVA).
