# Supplementary material for: The diadenosine tetraphosphate hydrolase ApaH contributes to Pseudomonas aeruginosa pathogenicity
Source: PLoS Pathog. 2024 Aug 19;20(8):e1012486. doi: 10.1371/journal.ppat.1012486 (PMC11361744; doi:10.1371/journal.ppat.1012486)
Supplement: S2 Fig — (A) Intracellular levels of ATP, ADP, GDP and GTP in P. aeruginosa PAO1 and the apaH mutant cultured at 37°C in LB. (B) Intracellular levels of ATP, ADP, GDP and GTP in P. aeruginosa PAO1 and the apaH mutant carrying the empty plasmid pME6032 or the plasmid pMEapaH, cultured at 37°C in LB supplemented with 100 μM IPTG. Values are the mean (± standard deviation) of three biological replicates. Asterisks indicate a statistically significant difference (P < 0.05) with respect to PAO1 (panel A; unpaired t test) or PAO1 pME6032 (panel B; ANOVA). (PDF) [file ppat.1012486.s006.pdf]

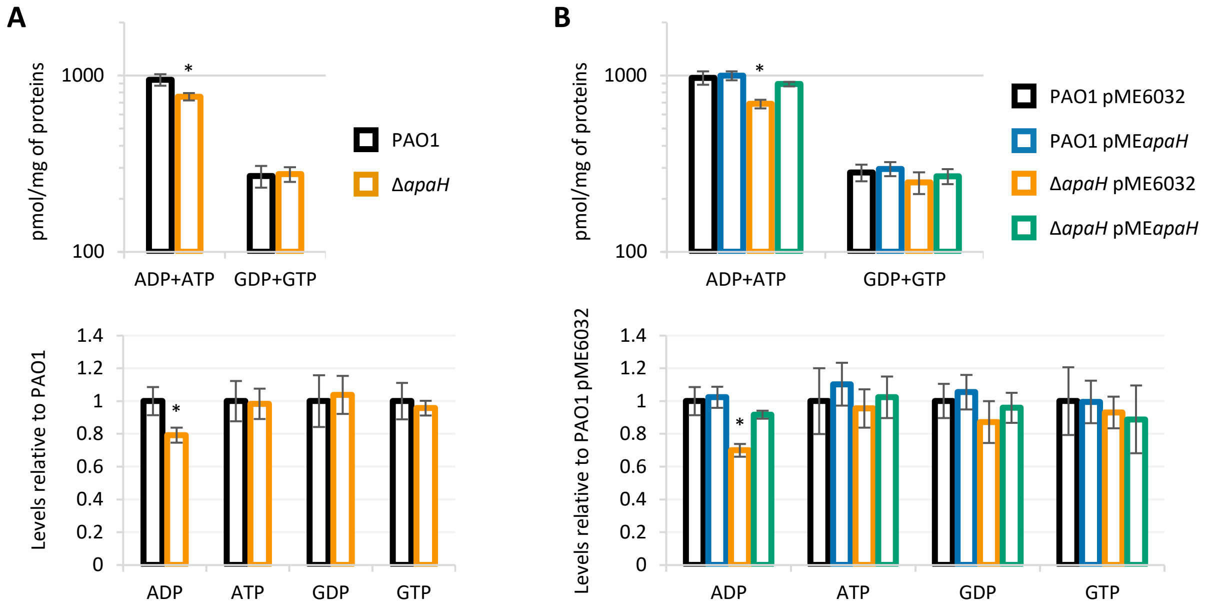

S2 Fig. (**A**) Intracellular levels of ATP, ADP, GDP and GTP in *P. aeruginosa* PAO1 and the *apaH* mutant cultured at 37°C in LB. (**B**) Intracellular levels of ATP, ADP, GDP and GTP in *P. aeruginosa* PAO1 and the *apaH* mutant carrying the empty plasmid pME6032 or the plasmid pME*apaH*, cultured at 37°C in LB supplemented with 100 µM IPTG. Values are the mean (± standard deviation) of three biological replicates. Asterisks indicate a statistically significant difference ($P < 0.05$) with respect to PAO1 (panel A; unpaired *t* test) or PAO1 pME6032 (panel B; ANOVA).
