# Supplementary material for: The diadenosine tetraphosphate hydrolase ApaH contributes to Pseudomonas aeruginosa pathogenicity
Source: PLoS Pathog. 2024 Aug 19;20(8):e1012486. doi: 10.1371/journal.ppat.1012486 (PMC11361744; doi:10.1371/journal.ppat.1012486)
Supplement: S3 Fig — Levels of the rhl QS signal molecule C4-HSL (A) and the las QS signal molecule 3OC12-HSL (B), normalized to cell density (OD600), in the supernatants of P. aeruginosa PAO1 and the apaH mutant, carrying or not the empty plasmid pME6032 or the plasmid pMEapaH, cultured at 37°C in LB, supplemented with 100 μM IPTG in the case of strains carrying the plasmids. Values are the mean (± standard deviation) of three independent assays. No statistically significant differences (P > 0.05) were observed with respect to PAO1 unpaired t test) or PAO1 pME6032 (ANOVA). (PDF) [file ppat.1012486.s007.pdf]

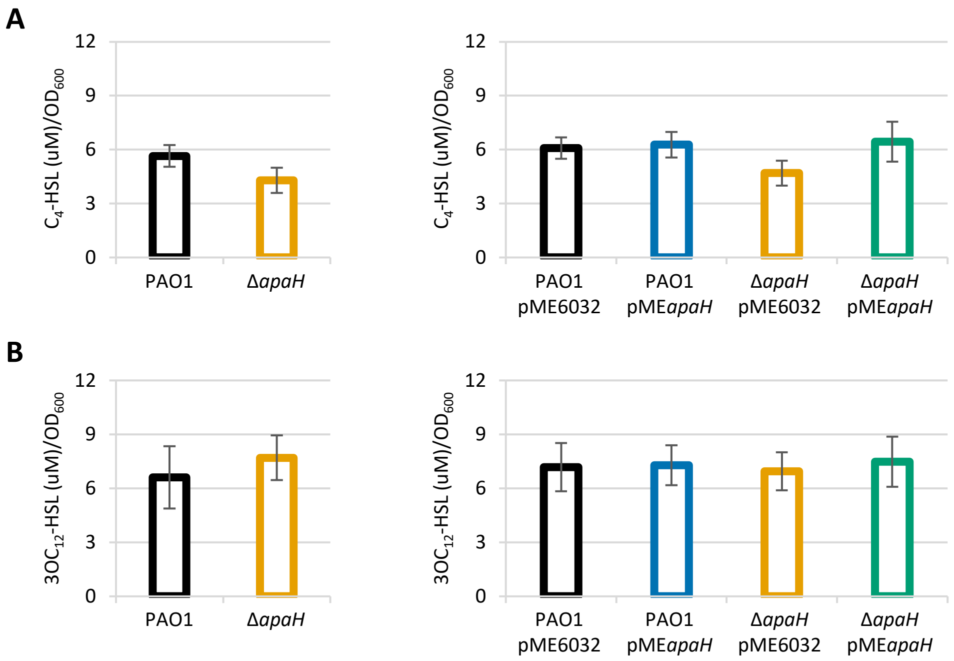

S3 Fig. Levels of the *rhl* QS signal molecule C_4-HSL (**A**) and the *las* QS signal molecule $3OC_{12}$-HSL (**B**), normalized to cell density (OD_{600}), in the supernatants of *P. aeruginosa* PAO1 and the *apaH* mutant, carrying or not the empty plasmid pME6032 or the plasmid pME*apaH*, cultured at 37°C in LB, supplemented with 100 µM IPTG in the case of strains carrying the plasmids. Values are the mean (± standard deviation) of three independent assays. No statistically significant differences ($P > 0.05$) were observed with respect to PAO1 unpaired *t* test) or PAO1 pME6032 (ANOVA).
