# Supplementary material for: The diadenosine tetraphosphate hydrolase ApaH contributes to Pseudomonas aeruginosa pathogenicity
Source: PLoS Pathog. 2024 Aug 19;20(8):e1012486. doi: 10.1371/journal.ppat.1012486 (PMC11361744; doi:10.1371/journal.ppat.1012486)
Supplement: S4 Fig — Values are the mean of three technical replicates and the curves are representative of three biological replicates. (PDF) [file ppat.1012486.s008.pdf]

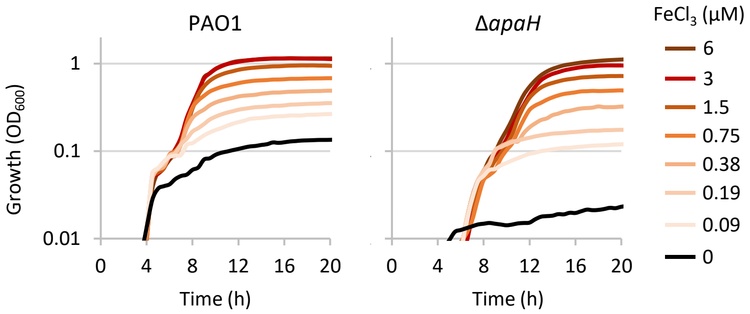

S4 Fig. Growth curves of *P. aeruginosa* PAO1 and the *apaH* mutant in the iron-poor medium CAA supplemented or not with the indicated concentrations of $FeCl_3$ in microtiter plates at 37°C in an automatic microtiter plate reader. Values are the mean of three technical replicates and the curves are representative of three biological replicates.
