# Supplementary material for: The diadenosine tetraphosphate hydrolase ApaH contributes to Pseudomonas aeruginosa pathogenicity
Source: PLoS Pathog. 2024 Aug 19;20(8):e1012486. doi: 10.1371/journal.ppat.1012486 (PMC11361744; doi:10.1371/journal.ppat.1012486)
Supplement: S5 Fig — Heat tolerance of (A) P. aeruginosa PAO1 and the apaH mutant or (B) the same strains carrying the empty plasmid pME6032 or the plasmid pMEapaH. Cells were cultured at 20°C in LB, supplemented with 100 μM IPTG for plasmid-harboring strains, and subjected to a heat shock at 50°C. Cell viability was monitored as CFU/mL at different time points. Values are the mean (± standard deviation) of at least three independent assays. Asterisks indicate a statistically significant difference (P < 0.05) with respect to PAO1 (panel A; unpaired t test) or PAO1 pME6032 (panel B; ANOVA). (PDF) [file ppat.1012486.s009.pdf]

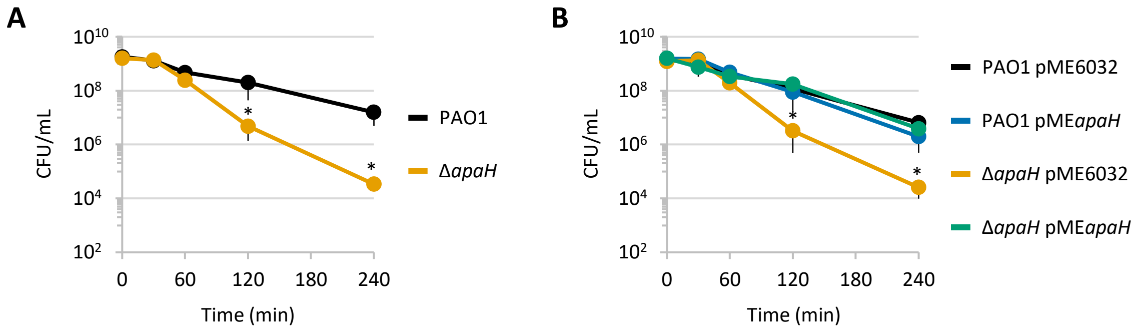

S5 Fig. Heat tolerance of (**A**) *P. aeruginosa* PAO1 and the *apaH* mutant or (**B**) the same strains carrying the empty plasmid pME6032 or the plasmid pME*apaH*. Cells were cultured at 20 °C in LB, supplemented with 100 µM IPTG for plasmid-harboring strains, and subjected to a heat shock at 50 °C. Cell viability was monitored as CFU/mL at different time points. Values are the mean (± standard deviation) of at least three independent assays. Asterisks indicate a statistically significant difference ($P < 0.05$) with respect to PAO1 (panel A; unpaired *t* test) or PAO1 pME6032 (panel B; ANOVA).
