# Supplementary material for: The diadenosine tetraphosphate hydrolase ApaH contributes to Pseudomonas aeruginosa pathogenicity
Source: PLoS Pathog. 2024 Aug 19;20(8):e1012486. doi: 10.1371/journal.ppat.1012486 (PMC11361744; doi:10.1371/journal.ppat.1012486)
Supplement: S6 Fig — (A) Protease activity, (B) elastase activity, and (C) PQS/HHQ levels, normalized to cell density (OD600), in the supernatants of P. aeruginosa PAO1 and the apaH mutant, carrying the empty plasmid pME6032 or the plasmid pMEapaH, cultured at 37°C in LB supplemented with 100 μM IPTG. (D) Growth curves and (E) pyoverdine levels, normalized to cell density (OD600), of the same strains cultured at 37°C in the iron-poor medium CAA, supplemented with 100 μM IPTG and 50 μM FeCl3 when indicated (+ Fe). Values are the mean (± standard deviation) of three independent assays. Asterisks indicate a statistically significant difference (P < 0.001) with respect to PAO1 pME6032 (ANOVA). (PDF) [file ppat.1012486.s010.pdf]

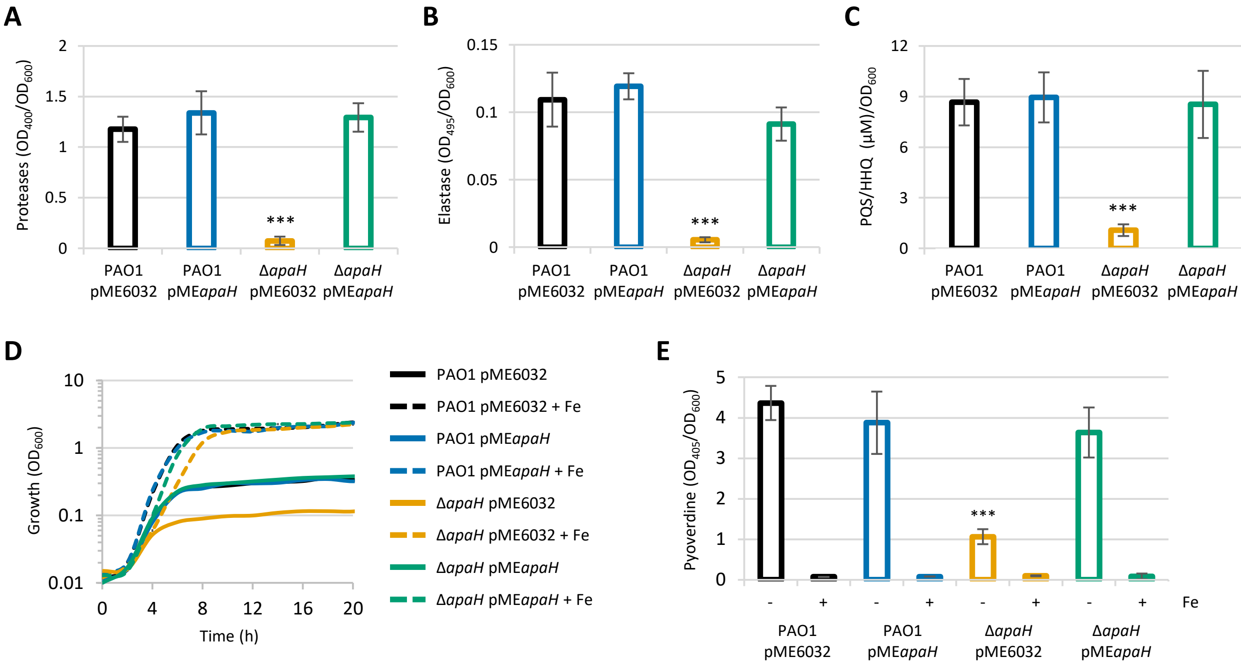

S6 Fig. (**A**) Protease activity, (**B**) elastase activity, and (**C**) PQS/HHQ levels, normalized to cell density (OD_{600}), in the supernatants of *P. aeruginosa* PAO1 and the *apaH* mutant, carrying the empty plasmid pME6032 or the plasmid pME*apaH*, cultured at 37°C in LB supplemented with 100 µM IPTG. (**D**) Growth curves and (**E**) pyoverdine levels, normalized to cell density (OD_{600}), of the same strains cultured at 37°C in the iron-poor medium CAA, supplemented with 100 µM IPTG and 50 µM $FeCl_3$ when indicated (+ Fe). Values are the mean (± standard deviation) of three independent assays. Asterisks indicate a statistically significant difference ($P < 0.001$) with respect to PAO1 pME6032 (ANOVA).
