# Supplementary material for: The diadenosine tetraphosphate hydrolase ApaH contributes to Pseudomonas aeruginosa pathogenicity
Source: PLoS Pathog. 2024 Aug 19;20(8):e1012486. doi: 10.1371/journal.ppat.1012486 (PMC11361744; doi:10.1371/journal.ppat.1012486)
Supplement: S7 Fig — Sixteen larvae were infected with each strain in two independent experiments. Asterisks indicate a statistically significant difference (P < 0.001) with respect to PAO1 (Mantel-Cox test). (PDF) [file ppat.1012486.s011.pdf]

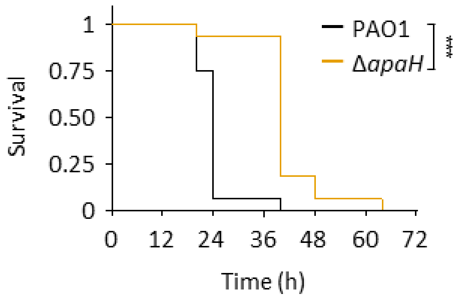

S7 Fig. Kaplan-Meier survival curves of *G. mellonella* larvae infected with 13.8 (± 2.8) PAO1 cells or 13.9 (± 0.2) Δ*apaH* cells. Sixteen larvae were infected with each strain in two independent experiments. Asterisks indicate a statistically significant difference ($P < 0.001$) with respect to PAO1 (Mantel-Cox test).
