# Supplementary material for: The diadenosine tetraphosphate hydrolase ApaH contributes to Pseudomonas aeruginosa pathogenicity
Source: PLoS Pathog. 2024 Aug 19;20(8):e1012486. doi: 10.1371/journal.ppat.1012486 (PMC11361744; doi:10.1371/journal.ppat.1012486)
Supplement: S8 Fig — (A) Relative mRNA levels of selected virulence genes (prpL, pvdD, aprA, lasA, lasB), determined by RT-qPCR, in P. aeruginosa PAO1 carrying the empty plasmid pME6032 or the plasmid pMEapaH, cultured in LB supplemented with 100 μM IPTG until mid-exponential phase. Values are the mean (± standard deviation) of three biological replicates. (B) Pathogenicity in the lettuce leaf virulence assay of PAO1 pME6032 and PAO1 pMEapaH precultured in LB supplemented with 100 μM IPTG. Bacterial viable cells (CFUs) per mg of lettuce midribs at three days post injection are shown. Five biological replicates for each strain were analyzed. Representative pictures of the infected midribs are shown. (C) Dose-dependent survival curves of G. mellonella larvae infected with different doses of PAO1 pME6032 or PAO1 pMEapaH precultured in LB supplemented with 100 μM IPTG. Lethal dose 90% (LD90) and R2 values are shown in the figure. (PDF) [file ppat.1012486.s012.pdf]

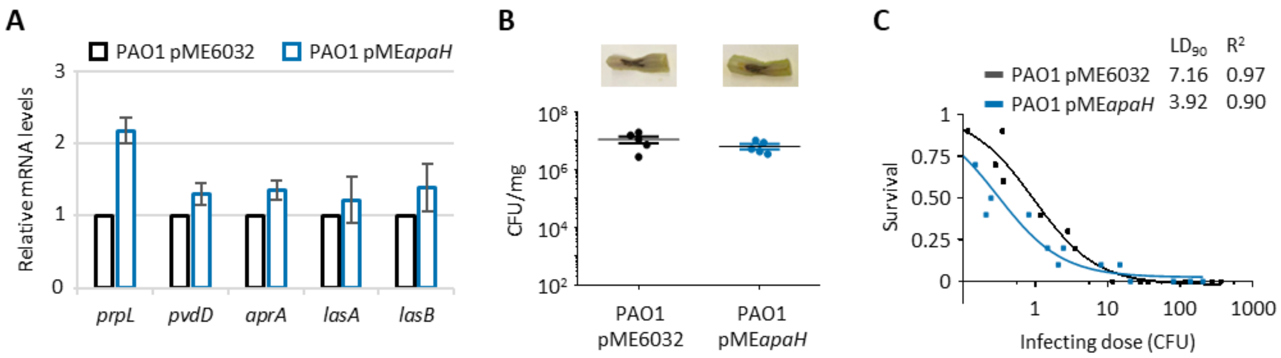

S8 Fig. (**A**) Relative mRNA levels of selected virulence genes (*prpL*, *pvdD*, *aprA*, *lasA*, *lasB*), determined by RT-qPCR, in *P. aeruginosa* PAO1 carrying the empty plasmid pME6032 or the plasmid pME*apaH*, cultured in LB supplemented with 100 µM IPTG until mid-exponential phase. Values are the mean (± standard deviation) of three biological replicates. (**B**) Pathogenicity in the lettuce leaf virulence assay of PAO1 pME6032 and PAO1 pME*apaH* precultured in LB supplemented with 100 µM IPTG. Bacterial viable cells (CFUs) per mg of lettuce midribs at three days post injection are shown. Five biological replicates for each strain were analyzed. Representative pictures of the infected midribs are shown. (**C**) Dose-dependent survival curves of *G. mellonella* larvae infected with different doses of PAO1 pME6032 or PAO1 pME*apaH* precultured in LB supplemented with 100 µM IPTG. Lethal dose 90% (LD_{90}) and R^2 values are shown in the figure.
