# Supplementary material for: The diadenosine tetraphosphate hydrolase ApaH contributes to Pseudomonas aeruginosa pathogenicity
Source: PLoS Pathog. 2024 Aug 19;20(8):e1012486. doi: 10.1371/journal.ppat.1012486 (PMC11361744; doi:10.1371/journal.ppat.1012486)
Supplement: S9 Fig — Values are the mean (± standard deviation) of three independent assays. The asterisk indicates a statistically significant difference (P < 0.05) between the ΔapaH mutant and its parental strain C1 (unpaired t test). (PDF) [file ppat.1012486.s013.pdf]

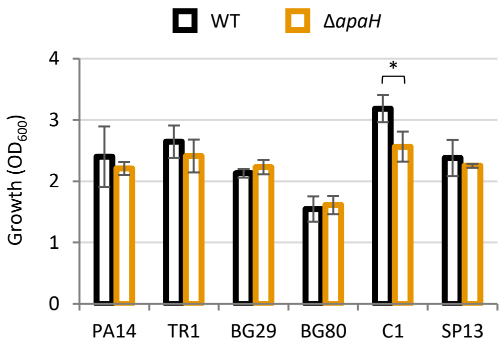

S9 Fig. Maximum growth yields of *P. aeruginosa* PA14 and the indicated *P. aeruginosa* clinical isolates (WT) or the cognate *apaH* mutants (Δ*apaH*) in CAA medium supplemented with 50 µM $FeCl_3$ over 24 h of growth at 37°C. Values are the mean (± standard deviation) of three independent assays. The asterisk indicates a statistically significant difference ($P < 0.05$) between the Δ*apaH* mutant and its parental strain C1 (unpaired *t* test).
