# Supplementary material for: The diadenosine tetraphosphate hydrolase ApaH contributes to Pseudomonas aeruginosa pathogenicity
Source: PLoS Pathog. 2024 Aug 19;20(8):e1012486. doi: 10.1371/journal.ppat.1012486 (PMC11361744; doi:10.1371/journal.ppat.1012486)
Supplement: S10 Fig — Lethal dose 90% (LD90) and R2 values are shown in the figure. (PDF) [file ppat.1012486.s014.pdf]

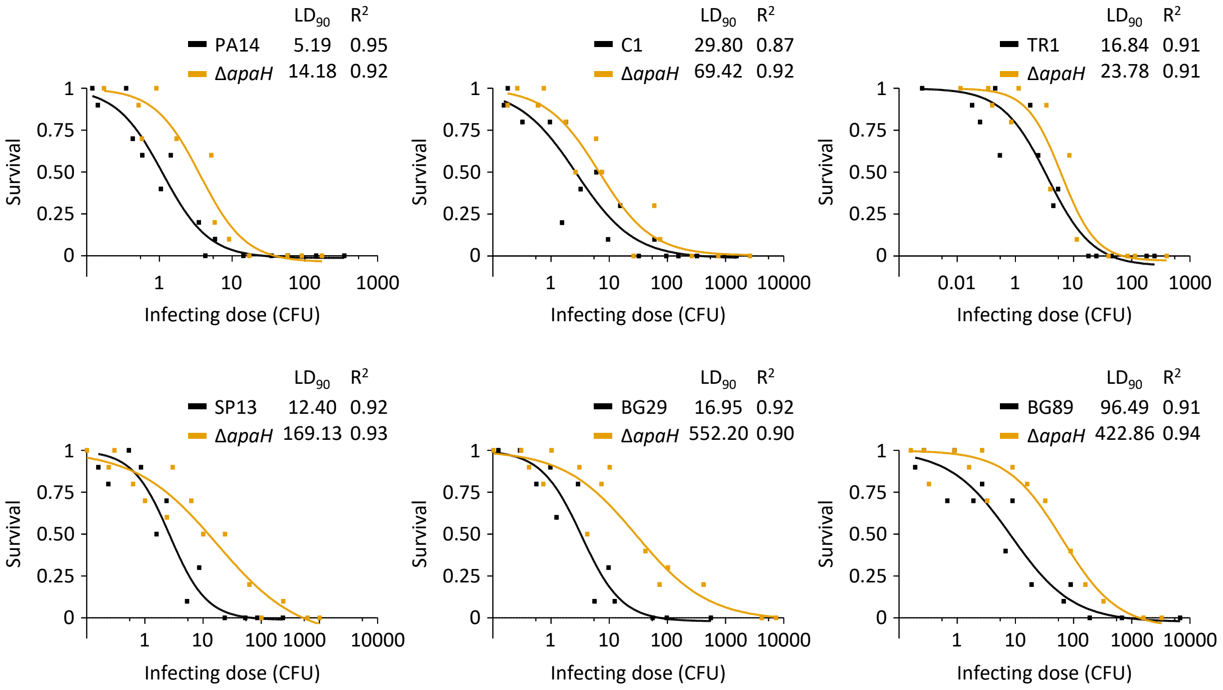

S10 Fig. Dose-dependent survival curves of *G. mellonella* larvae infected with different doses of the indicated wild type strains (black lines and symbols) or the corresponding Δ*apaH* mutants (orange lines and symbols). Lethal dose 90% (LD_{90}) and R^2 values are shown in the figure.
