# Supplementary material for: The diadenosine tetraphosphate hydrolase ApaH contributes to Pseudomonas aeruginosa pathogenicity
Source: PLoS Pathog. 2024 Aug 19;20(8):e1012486. doi: 10.1371/journal.ppat.1012486 (PMC11361744; doi:10.1371/journal.ppat.1012486)
Supplement: S1 Data — (PDF) [file ppat.1012486.s015.pdf]

Figure 1A

Strain	OD600													
	0	2	4	6	8	10	12	14	16	18	20	22	24	Time (h)
PAO1 1	0.00	0.11	0.94	1.98	2.56	2.81	2.80	3.09	3.30	3.52	3.72	3.62	3.58	
PAO1 2	0.00	0.11	0.99	2.07	2.59	2.88	3.09	3.26	3.41	3.55	3.53	3.71	3.92	
PAO1 3	0.00	0.08	0.77	1.94	2.34	2.70	3.10	3.25	3.47	3.72	3.76	3.74	3.75	
PAO1 ΔapaH 1	0.00	0.04	0.26	1.00	1.60	1.65	1.60	1.39	1.39	1.46	1.26	1.26	1.23	
PAO1 ΔapaH 2	0.00	0.06	0.47	1.08	1.65	1.46	1.47	1.55	1.38	1.36	1.49	1.41	1.48	
PAO1 ΔapaH 3	0.00	0.07	0.57	1.21	1.49	1.52	1.63	1.36	1.54	1.50	1.55	1.62	1.59	
PAO1 ΔapaH pMEapaH 1	0.00	0.10	0.84	1.94	2.39	2.57	2.88	3.07	3.15	3.27	3.60	3.62	3.56	
PAO1 ΔapaH pMEapaH 2	0.00	0.09	0.90	2.00	2.51	2.84	2.60	3.01	3.19	3.50	3.55	3.32	3.28	
PAO1 ΔapaH pMEapaH 3	0.00	0.09	0.88	1.72	2.16	2.45	2.88	2.72	2.89	3.18	3.34	3.49	3.66	

Strain	AVERAGE													
	0	2	4	6	8	10	12	14	16	18	20	22	24	Time (h)
PAO1	0.00	0.10	0.90	2.00	2.50	2.80	3.00	3.20	3.39	3.60	3.67	3.69	3.75	
PAO1 ΔapaH	0.00	0.05	0.43	1.10	1.58	1.54	1.57	1.43	1.44	1.44	1.43	1.43	1.43	
PAO1 ΔapaH pMEapaH	0.00	0.09	0.87	1.89	2.35	2.62	2.79	2.93	3.08	3.31	3.50	3.48	3.50	

Strain	SD													
	0	2	4	6	8	10	12	14	16	18	20	22	24	Time (h)
PAO1	0.00	0.02	0.12	0.07	0.14	0.09	0.17	0.10	0.09	0.11	0.12	0.06	0.17	
PAO1 ΔapaH	0.00	0.01	0.16	0.11	0.08	0.10	0.08	0.10	0.09	0.07	0.15	0.18	0.18	
PAO1 ΔapaH pMEapaH	0.00	0.00	0.03	0.15	0.18	0.20	0.16	0.19	0.17	0.17	0.14	0.15	0.20	

Figure 1B

Strain	Replicate	Ap4A (pmol/mg of proteins)
PAO1	1	9.9
PAO1 ΔapaH	1	166.8
PAO1	2	5.3
PAO1 ΔapaH	2	252.7
PAO1	3	9.9
PAO1 ΔapaH	3	219.6

	Ap4A (pmol) per mg of protein	
	Average	SD
PAO1	8.4	2.66
PAO1 ΔapaH	213.0	43.29

Figure 1C

Strain	OD595/OD600
PAO1 1	0.67
PAO1 2	0.65
PAO1 3	0.61
PAO1 4	0.98
PAO1 5	0.94
PAO1 6	1.16
PAO1 ΔapaH 1	0.67
PAO1 ΔapaH 2	0.70
PAO1 ΔapaH 3	0.68
PAO1 ΔapaH 4	0.88
PAO1 ΔapaH 5	0.92
PAO1 ΔapaH 6	1.00

Strain	OD595/OD600	
	Average	SD
PAO1	0.83	0.22
PAO1 ΔapaH	0.81	0.14

Figure 1D

Strain	Replicate	c-di-GMP (pmol/mg of proteins)
PAO1	1	18.6
PAO1 ΔapaH	1	17.1
PAO1	2	21.0
PAO1 ΔapaH	2	21.1
PAO1	3	15.6
PAO1 ΔapaH	3	14.4

	c-di-GMP (pmol) per mg of protein	
	Average	SD
PAO1	18.4	2.72
PAO1 ΔapaH	17.5	3.37

Figure 2A

Strain	Replicate	Inhibition halos (mm)							
		**Sm**	**Gm**	**NN**	**Cip**	**Imp**	**Ery**	**NB**	**Rif**
PAO1	1	12	24	25	30	24	6	6	6
PAO1 ΔapaH		11	25	25	30	25	6	6	6
PAO1	2	15	25	26	31	25	6	6	6
PAO1 ΔapaH		13	25	25	31	26	6	6	6
PAO1	3	16	23	25	33	26	6	6	6
PAO1 ΔapaH		17	25	25	34	27	6	7	6
PAO1	4	17		25				6	
PAO1 ΔapaH		17		28				8	
PAO1	5	16		24				6	
PAO1 ΔapaH		17		30				7	

AVERAGE		**Sm**	**Gm**	**NN**	**Cip**	**Imp**	**Ery**	**NB**	**Rif**
PAO1		15.2	24.0	25.0	31.3	25.0	6.0	6.0	6.0
PAO1 ΔapaH		15.0	25.0	26.6	31.7	26.0	6.0	6.8	6.0

SD		**Sm**	**Gm**	**NN**	**Cip**	**Imp**	**Ery**	**NB**	**Rif**
PAO1		1.9	1.0	0.7	1.5	1.0	0.0	0.0	0.0
PAO1 ΔapaH		2.8	0.0	2.3	2.1	1.0	0.0	0.8	0.0

Figure 2B

Replicate 1		CFU/mL				
Gm (µg/mL)	Strain	0	1	2	4	24
0.5	PAO1	6.50E+05	7.50E+03	1.50E+02	5.00E+01	2.50E+01
	PAO1 ΔapaH	3.50E+05	1.40E+04	2.50E+02	2.50E+01	2.50E+01
1	PAO1	6.50E+05	5.50E+02	5.00E+01	2.50E+01	2.50E+01
	PAO1 ΔapaH	3.50E+05	7.50E+02	2.50E+01	2.50E+01	2.50E+01

Time (h)

AVERAGE						
Gm (µg/mL)	Strain	0	1	2	4	24
0.5	PAO1	5.90E+05	8.14E+04	2.00E+02	6.67E+01	2.50E+01
	PAO1 ΔapaH	4.80E+05	1.20E+04	2.83E+02	1.83E+02	2.50E+01
1	PAO1	5.90E+05	2.37E+03	5.00E+01	2.50E+01	2.50E+01
	PAO1 ΔapaH	4.80E+05	1.30E+03	5.00E+01	2.50E+01	2.50E+01

Replicate 2		CFU/mL				
Gm (µg/mL)	Strain	0	1	2	4	24
0.5	PAO1	7.00E+05	2.30E+05	2.50E+02	5.00E+01	2.50E+01
	PAO1 ΔapaH	6.00E+05	1.85E+04	3.50E+02	2.50E+01	2.50E+01
1	PAO1	7.00E+05	6.00E+03	5.00E+01	2.50E+01	2.50E+01
	PAO1 ΔapaH	6.00E+05	2.10E+03	1.00E+02	2.50E+01	2.50E+01

Time (h)

SD						
Gm (µg/mL)	Strain	0	1	2	4	24
0.5	PAO1	1.49.E+05	1.29.E+05	5.00.E+01	2.89.E+01	0.00.E+00
	PAO1 ΔapaH	1.25.E+05	7.75.E+03	5.77.E+01	2.74.E+02	0.00.E+00
1	PAO1	1.49.E+05	3.15.E+03	0.00.E+00	0.00.E+00	0.00.E+00
	PAO1 ΔapaH	1.25.E+05	7.09.E+02	4.33.E+01	0.00.E+00	0.00.E+00

Replicate 3		CFU/mL				
Gm (µg/mL)	Strain	0	1	2	4	24
0.5	PAO1	4.20E+05	6.75E+03	2.00E+02	1.00E+02	2.50E+01
	PAO1 ΔapaH	4.90E+05	3.40E+03	2.50E+02	5.00E+02	2.50E+01
1	PAO1	4.20E+05	5.50E+02	5.00E+01	2.50E+01	2.50E+01
	PAO1 ΔapaH	4.90E+05	1.05E+03	2.50E+01	2.50E+01	2.50E+01

Time (h)

Replicate 1		CFU/mL				
Km (µg/mL)	Strain	0	1	2	4	24
32	PAO1	4.45E+05	4.90E+04	1.85E+04	3.65E+03	2.50E+01
	PAO1 ΔapaH	4.15E+05	2.30E+05	5.05E+04	4.65E+04	4.00E+02
64	PAO1	4.45E+05	4.20E+03	4.00E+02	1.50E+02	2.50E+01
	PAO1 ΔapaH	4.15E+05	3.50E+04	4.50E+03	2.10E+03	2.50E+01

Time (h)

AVERAGE						
Km (µg/mL)	Strain	0	1	2	4	24
32	PAO1	7.82E+05	7.30E+04	1.21E+04	2.00E+03	2.50E+01
	PAO1 ΔapaH	4.48E+05	1.62E+05	3.85E+04	2.47E+04	2.08E+02
64	PAO1	7.82E+05	7.67E+03	4.67E+02	1.50E+02	2.50E+01
	PAO1 ΔapaH	4.48E+05	2.05E+04	2.37E+03	1.04E+03	2.50E+01

Replicate 2		CFU/mL				
Km (µg/mL)	Strain	0	1	2	4	24
32	PAO1	9.00E+05	3.00E+04	5.85E+03	7.50E+02	2.50E+01
	PAO1 ΔapaH	3.80E+05	4.65E+04	3.00E+04	4.65E+03	2.50E+01
64	PAO1	9.00E+05	3.30E+03	7.00E+02	5.00E+01	2.50E+01
	PAO1 ΔapaH	3.80E+05	6.50E+03	1.45E+03	2.50E+01	2.50E+01

Time (h)

SD						
Km (µg/mL)	Strain	0	1	2	4	24
32	PAO1	2.96.E+05	5.88.E+04	6.33.E+03	1.49.E+03	0.00.E+00
	PAO1 ΔapaH	8.98.E+04	1.01.E+05	1.07.E+04	2.10.E+04	1.88.E+02
64	PAO1	2.96.E+05	6.80.E+03	2.08.E+02	1.00.E+02	0.00.E+00
	PAO1 ΔapaH	8.98.E+04	1.43.E+04	1.85.E+03	1.04.E+03	0.00.E+00

Replicate 3		CFU/mL				
Km (µg/mL)	Strain	0	1	2	4	24
32	PAO1	1.00E+06	1.40E+05	1.20E+04	1.60E+03	2.50E+01
	PAO1 ΔapaH	5.50E+05	2.10E+05	3.50E+04	2.30E+04	2.00E+02
64	PAO1	1.00E+06	1.55E+04	3.00E+02	2.50E+02	2.50E+01
	PAO1 ΔapaH	5.50E+05	2.00E+04	1.15E+03	1.00E+03	2.50E+01

Time (h)

Figure 3

PseudoCAP	DEGs (no)	Downregulated genes (no.)	Upregulated genes (no.)	Total (no.)	Downregulated genes (%)	Upregulated genes (%)
Hypothetical, unclassified, unknown	60	38	22	1884	2.0	1.2
Transport of small molecules	30	27	3	609	4.4	0.5
Translation, post-translational modification, degradation	29	28	1	198	14.1	0.5
Secreted Factors (toxins, enzymes, alginate)	22	22	0	104	21.2	0.0
Membrane proteins	21	14	7	675	2.1	1.0
Energy metabolism	20	10	10	206	4.9	4.9
Adaptation, Protection	17	15	2	208	7.2	1.0
Putative enzymes	11	8	3	472	1.7	0.6
Central intermediary metabolism	9	8	1	108	7.4	0.9
Chaperones & heat shock proteins	9	9	0	56	16.1	0.0
Carbon compound catabolism	6	4	2	193	2.1	1.0
Motility & Attachment	6	6	0	141	4.3	0.0
Amino acid biosynthesis and metabolism	5	5	0	262	1.9	0.0
Biosynthesis of cofactors, prosthetic groups and carriers	5	5	0	160	3.1	0.0
Antibiotic resistance and susceptibility	4	3	1	74	4.1	1.4
Non-coding RNA gene	3	3	0	110	2.7	0.0
Cell division	2	2	0	30	6.7	0.0
DNA replication, recombination, modification and repair	2	2	0	88	2.3	0.0
Transcription, RNA processing and degradation	2	2	0	56	3.6	0.0
Transcriptional regulators	2	2	0	490	0.4	0.0
Fatty acid and phospholipid metabolism	1	1	0	64	1.6	0.0
Protein secretion/export apparatus	1	0	1	142	0.0	0.7

Figure 4A

Strain	OD400/OD600
PAO1 1	0.85
PAO1 2	1.20
PAO1 3	1.24
PAO1 ΔapaH 1	0.07
PAO1 ΔapaH 2	0.16
PAO1 ΔapaH 3	0.15
PAO1 ΔapaH pMEapaH 1	1.14
PAO1 ΔapaH pMEapaH 2	1.42
PAO1 ΔapaH pMEapaH 3	1.32

	OD400/OD600	
	Average	SD
PAO1	1.09	0.22
PAO1 ΔapaH	0.12	0.05
PAO1 ΔapaH pMEapaH	1.29	0.14

Figure 4B

Strain	OD495/OD600
PAO1 1	0.05
PAO1 2	0.07
PAO1 3	0.08
PAO1 ΔapaH 1	0.00
PAO1 ΔapaH 2	0.00
PAO1 ΔapaH 3	0.00
PAO1 ΔapaH pMEapaH 1	0.08
PAO1 ΔapaH pMEapaH 2	0.11
PAO1 ΔapaH pMEapaH 3	0.09

	OD495/OD600	
	Average	SD
PAO1	0.07	0.02
PAO1 ΔapaH	0.00	0.00
PAO1 ΔapaH pMEapaH	0.09	0.01

Figure 4C

Strain	PQS (μM)/OD600
PAO1 1	8.65
PAO1 2	5.56
PAO1 3	9.93
PAO1 4	8.51
PAO1 ΔapaH 1	0.43
PAO1 ΔapaH 2	0.89
PAO1 ΔapaH 3	1.34
PAO1 ΔapaH 4	1.23
PAO1 ΔapaH pMEapaH 1	6.10
PAO1 ΔapaH pMEapaH 2	9.39
PAO1 ΔapaH pMEapaH 3	10.74
PAO1 ΔapaH pMEapaH 4	7.93

	PQS (μM)/OD600	
	Average	SD
PAO1	8.16	1.85
PAO1 ΔapaH	0.97	0.41
PAO1 ΔapaH pMEapaH	8.54	1.99

Figure 4D

Replicate 1	OD600													
	0	2	4	6	8	10	12	14	16	18	20	22	24	Time (h)
PAO1	0.001	0.016	0.132	0.252	0.264	0.304	0.244	0.288	0.336	0.312	0.288	0.288	0.304	
PAO1 + Fe	0.001	0.014	0.182	0.998	1.868	1.832	2.020	2.098	2.176	2.280	2.320	2.420	2.498	
ΔapaH	0.001	0.008	0.036	0.062	0.058	0.066	0.088	0.100	0.108	0.108	0.088	0.080	0.084	
ΔapaH + Fe	0.001	0.012	0.036	0.244	1.142	1.362	1.444	1.340	1.740	2.020	2.124	2.226	2.210	
ΔapaH pMEapaH	0.001	0.012	0.112	0.288	0.296	0.312	0.304	0.316	0.352	0.368	0.320	0.348	0.356	
ΔapaH pMEapaH + Fe	0.001	0.015	0.132	0.744	1.640	1.818	1.886	2.128	2.544	2.388	2.160	2.420	2.414	

Replicate 2	OD600													
	0	2	4	6	8	10	12	14	16	18	20	22	24	Time (h)
PAO1	0.001	0.008	0.092	0.216	0.288	0.316	0.284	0.308	0.320	0.320	0.360	0.344	0.325	
PAO1 + Fe	0.001	0.012	0.156	1.120	2.208	2.310	2.380	2.426	2.324	2.326	2.480	2.490	2.512	
ΔapaH	0.001	0.000	0.020	0.060	0.068	0.075	0.084	0.100	0.108	0.100	0.108	0.100	0.095	
ΔapaH + Fe	0.001	0.000	0.024	0.224	1.336	1.812	1.784	1.968	1.878	1.996	2.030	2.092	2.096	
ΔapaH pMEapaH	0.001	0.005	0.079	0.188	0.232	0.268	0.321	0.338	0.344	0.368	0.388	0.396	0.385	
ΔapaH pMEapaH + Fe	0.001	0.006	0.088	0.824	1.992	2.132	2.296	2.176	2.144	2.392	2.504	2.448	2.480	

Replicate 3	OD600													
	0	2	4	6	8	10	12	14	16	18	20	22	24	Time (h)
PAO1	0.001	0.007	0.088	0.200	0.252	0.328	0.272	0.312	0.328	0.332	0.356	0.360	0.340	
PAO1 + Fe	0.001	0.009	0.139	1.032	2.312	2.522	2.494	2.532	2.512	2.466	2.502	2.476	2.446	
ΔapaH	0.001	0.000	0.040	0.064	0.076	0.079	0.082	0.096	0.096	0.100	0.096	0.104	0.080	
ΔapaH + Fe	0.001	0.000	0.048	0.260	1.549	1.882	1.958	2.098	1.964	1.836	2.104	2.220	2.256	
ΔapaH pMEapaH	0.001	0.004	0.086	0.204	0.316	0.326	0.312	0.352	0.348	0.372	0.364	0.404	0.425	
ΔapaH pMEapaH + Fe	0.001	0.008	0.112	0.804	2.116	2.364	2.404	2.352	2.084	2.184	2.428	2.552	2.510	

	AVERAGE													
	0	2	4	6	8	10	12	14	16	18	20	22	24	Time (h)
PAO1	0.001	0.010	0.104	0.223	0.268	0.316	0.267	0.303	0.328	0.321	0.335	0.331	0.323	
PAO1 + Fe	0.001	0.012	0.159	1.050	2.129	2.221	2.298	2.352	2.337	2.357	2.434	2.462	2.485	
ΔapaH	0.001	0.003	0.032	0.062	0.067	0.073	0.085	0.099	0.104	0.103	0.097	0.095	0.086	
ΔapaH + Fe	0.001	0.004	0.036	0.243	1.342	1.685	1.729	1.802	1.861	1.951	2.086	2.179	2.187	
ΔapaH pMEapaH	0.001	0.007	0.092	0.227	0.281	0.302	0.312	0.335	0.348	0.369	0.357	0.383	0.389	
ΔapaH pMEapaH + Fe	0.001	0.010	0.111	0.791	1.916	2.105	2.195	2.219	2.257	2.321	2.364	2.473	2.468	

	SD													
	0	2	4	6	8	10	12	14	16	18	20	22	24	Time (h)
PAO1	0.000	0.005	0.024	0.027	0.018	0.012	0.021	0.013	0.008	0.010	0.040	0.038	0.018	
PAO1 + Fe	0.000	0.003	0.022	0.063	0.232	0.353	0.247	0.226	0.168	0.097	0.099	0.037	0.035	
ΔapaH	0.000	0.005	0.011	0.002	0.009	0.007	0.003	0.002	0.007	0.005	0.010	0.013	0.008	
ΔapaH + Fe	0.000	0.007	0.012	0.018	0.203	0.282	0.261	0.405	0.113	0.100	0.050	0.076	0.082	
ΔapaH pMEapaH	0.000	0.004	0.017	0.054	0.044	0.030	0.009	0.018	0.004	0.002	0.034	0.030	0.035	
ΔapaH pMEapaH + Fe	0.000	0.005	0.022	0.042	0.247	0.274	0.273	0.118	0.250	0.119	0.181	0.070	0.049	

Figure 4E

Strain	OD405/OD600	
	CAA	CAA+Fe
PAO1 1	4.34	0.07
PAO1 2	4.33	0.32
PAO1 3	3.72	0.08
PAO1 4	4.58	0.02
PAO1 5	4.08	0.03
PAO1 6	4.23	0.04
PAO1 ΔapaH 1	1.95	0.14
PAO1 ΔapaH 2	1.59	0.12
PAO1 ΔapaH 3	1.25	0.03
PAO1 ΔapaH 4	1.82	0.02
PAO1 ΔapaH 5	1.33	0.05
PAO1 ΔapaH 6	1.39	0.05
PAO1 ΔapaH pMEapaH 1	3.52	0.09
PAO1 ΔapaH pMEapaH 2	4.41	0.21
PAO1 ΔapaH pMEapaH 3	3.79	0.11
PAO1 ΔapaH pMEapaH 4	4.16	0.02
PAO1 ΔapaH pMEapaH 5	3.20	0.04
PAO1 ΔapaH pMEapaH 6	2.74	0.04

	OD405/OD600			
	Average		SD	
	CAA	CAA+Fe	CAA	CAA+Fe
PAO1	4.21	0.09	0.29	0.12
PAO1 ΔapaH	1.55	0.07	0.28	0.05
PAO1 ΔapaH pMEapaH	3.64	0.08	0.62	0.07

Figure 5A

Strain	Replicate	No. of CFUs in 1 mg of lettuce
PAO1	1	2.45E+07
	2	1.90E+07
	3	1.00E+06
	4	2.75E+06
	5	1.15E+06
	6	1.15E+06
	7	3.10E+06
	8	2.60E+06
	9	1.75E+06
	10	2.00E+06
PAO1 ΔapaH	1	5.00E+04
	2	8.00E+04
	3	3.00E+04
	4	4.00E+04
	5	1.65E+05
	6	5.00E+03
	7	2.65E+05
	8	8.50E+04
	9	1.80E+05
	10	1.85E+05

Figure 5B

PAO1	
Infecting dose (CFUs)	Survival
48	0
35	0
16	0
12	0
4.8	0
3.5	0.125
1.6	0.125
1.2	0.25
0.48	0.75
0.35	0.75
0.16	1
0.12	1

PAO1 ΔapaH	
Infecting dose (CFUs)	Survival
42	0
41	0
14	0
14	0
4.2	0.25
4.1	0.25
1.4	0.625
1.4	0.625
0.42	0.75
0.41	0.75
0.14	1
0.14	1

Figure 5C

DPI	PAO1	ΔapaH
1	1	
1	1	
1	1	
1	1	
1	1	1
5		0
5		0
5		0
5		0

Legend	
DPI	days post infection
1	censored
0	non censored

Figure 5D

CFUs in Lungs	
PAO1	ΔapaH
2.40E+04	4.90E+06
6.80E+09	1.12E+03
7.28E+09	1.72E+05
2.12E+08	6.30E+02
7.66E+06	1.16E+03

CFUs in Spleen	
PAO1	ΔapaH
7.38E+06	3.00E+02
2.74E+07	3.30E+02
9.24E+04	3.46E+03
4.50E+03	2.79E+03
3.76E+06	1.56E+03

Figure 5E

Total Leukocytes	
PAO1	ΔapaH
5.69E+05	1.03E+06
1.13E+06	1.30E+06
3.42E+05	1.22E+05
2.04E+05	6.46E+04
5.90E+04	1.35E+05

PMN in BAL	
PAO1	ΔapaH
5.38E+05	9.03E+05
1.04E+06	6.51E+05
5.36E+05	1.10E+05
2.04E+05	1.69E+04
2.88E+04	1.29E+05

Macrophages	
PAO1	ΔapaH
5.74E+04	4.60E+04
2.33E+05	1.84E+05
9.28E+04	1.45E+04
2.85E+04	4.70E+04
2.82E+04	9.87E+04

Lymphocytes	
PAO1	ΔapaH
5.74E+04	2.54E+05
7.06E+04	2.96E+04
2.22E+04	1.55E+04
3.21E+03	3.22E+04
1.66E+04	9.60E+02

Figure 6A

		OD400/OD600	
		WT	ΔapaH
Replicate 1	PA14	0.84	0.16
	TR1	0.00	0.00
	BG29	0.62	0.16
	BG80	0.20	0.01
	C1	0.11	0.00
	SP13	0.01	0.00
Replicate 2	PA14	0.85	0.32
	TR1	0.00	0.00
	BG29	0.78	0.00
	BG80	0.26	0.02
	C1	0.17	0.01
	SP13	0.01	0.01
Replicate 3	PA14	1.17	0.12
	TR1	0.00	0.00
	BG29	0.98	0.01
	BG80	0.26	0.00
	C1	0.21	0.01
	SP13	0.00	0.00

	Average		SD	
	WT	ΔapaH	WT	ΔapaH
PA14	0.96	0.20	0.19	0.11
TR1	0.00	0.00	0.00	0.00
BG29	0.79	0.06	0.18	0.09
BG80	0.24	0.01	0.03	0.01
C1	0.17	0.01	0.05	0.01
SP13	0.01	0.00	0.00	0.00

Figure 6B

		OD495/OD600	
		WT	ΔapaH
Replicate 1	PA14	0.08	0.01
	TR1	0.00	0.00
	BG29	0.08	0.04
	BG80	0.00	0.00
	C1	0.00	0.00
	SP13	0.00	0.00
Replicate 2	PA14	0.09	0.02
	TR1	0.00	0.00
	BG29	0.12	0.02
	BG80	0.00	0.00
	C1	0.00	0.00
	SP13	0.00	0.00
Replicate 3	PA14	0.06	0.00
	TR1	0.00	0.00
	BG29	0.08	0.01
	BG80	0.00	0.00
	C1	0.00	0.00
	SP13	0.00	0.00

	Average		SD	
	WT	ΔapaH	WT	ΔapaH
PA14	0.08	0.01	0.01	0.01
TR1	0.00	0.00	0.00	0.00
BG29	0.10	0.02	0.02	0.01
BG80	0.00	0.00	0.00	0.00
C1	0.00	0.00	0.00	0.00
SP13	0.00	0.00	0.00	0.00

Figure 6C

		OD600	
		WT	ΔapaH
Replicate 1	PA14	0.30	0.18
	TR1	0.55	0.35
	BG29	0.32	0.11
	BG80	0.22	0.00
	C1	0.49	0.25
	SP13	0.43	0.15
Replicate 2	PA14	0.32	0.14
	TR1	0.44	0.29
	BG29	0.27	0.13
	BG80	0.26	0.00
	C1	0.51	0.26
	SP13	0.40	0.19
Replicate 3	PA14	0.30	0.15
	TR1	0.48	0.36
	BG29	0.36	0.15
	BG80	0.25	0.00
	C1	0.40	0.30
	SP13	0.36	0.11

	Average		SD	
	WT	ΔapaH	WT	ΔapaH
PA14	0.31	0.15	0.01	0.02
TR1	0.49	0.34	0.06	0.04
BG29	0.32	0.13	0.05	0.02
BG80	0.24	0.00	0.02	0.00
C1	0.47	0.27	0.06	0.03
SP13	0.39	0.15	0.03	0.04

Figure 6D

		OD405/OD600	
		WT	ΔapaH
Replicate 1	PA14	3.64	0.67
	TR1	1.07	0.51
	BG29	3.11	1.68
	BG80	3.05	0.00
	C1	3.94	0.73
	SP13	2.73	0.46
Replicate 2	PA14	3.16	0.85
	TR1	1.09	0.68
	BG29	2.38	2.98
	BG80	3.45	0.00
	C1	4.23	0.85
	SP13	3.68	0.42
Replicate 3	PA14	3.15	0.68
	TR1	0.99	0.46
	BG29	2.58	2.54
	BG80	4.20	0.00
	C1	4.82	0.41
	SP13	4.39	0.78

	Average		SD	
	WT	ΔapaH	WT	ΔapaH
PA14	3.31	0.74	0.28	0.10
TR1	1.05	0.55	0.05	0.11
BG29	2.69	2.40	0.37	0.66
BG80	3.57	0.00	0.58	0.00
C1	4.33	0.66	0.45	0.23
SP13	3.60	0.55	0.83	0.20

Figure 7A

Strain	Replicate	No. of CFUs in 1 mg of lettuce
PA14	1	1.10E+07
	2	8.10E+06
	3	5.80E+06
	4	1.20E+07
	5	4.00E+06
C1	1	3.20E+05
	2	5.70E+05
	3	9.20E+04
	4	6.40E+05
	5	4.60E+04
TR1	1	2.50E+04
	2	1.50E+04
	3	2.70E+04
	4	5.50E+04
	5	7.70E+03
SP13	1	4.50E+04
	2	5.50E+04
	3	6.60E+04
	4	1.30E+05
	5	2.30E+04
BG29	1	1.20E+06
	2	1.60E+06
	3	5.70E+06
	4	1.10E+07
	5	7.80E+05
BG80	1	5.80E+06
	2	4.40E+06
	3	4.40E+06
	4	1.20E+07
	5	2.20E+06

Strain	Replicate	No. of CFUs in 1 mg of lettuce
PA14 ΔapaH	1	6.50E+04
	2	6.70E+04
	3	1.90E+05
	4	1.30E+05
	5	3.20E+04
C1 ΔapaH	1	7.80E+03
	2	6.20E+04
	3	8.00E+04
	4	1.60E+04
	5	3.10E+04
TR1 ΔapaH	1	1.30E+03
	2	1.90E+04
	3	1.50E+03
	4	3.70E+04
	5	6.40E+02
SP13 ΔapaH	1	2.80E+04
	2	1.00E+04
	3	3.40E+04
	4	6.80E+04
	5	5.20E+03
BG29 ΔapaH	1	9.00E+03
	2	4.30E+04
	3	2.90E+04
	4	5.80E+04
	5	4.50E+03
BG80 ΔapaH	1	6.50E+03
	2	4.00E+04
	3	5.50E+04
	4	8.10E+04
	5	3.20E+03

Figure 7B

	LD90	R^2	Ratio (ΔapaH vs WT)
PA14	5.19	0.946	
PA14 ΔapaH	14.18	0.918	2.73
TR1	16.84	0.909	
TR1 ΔapaH	23.78	0.905	1.41
SP13	12.40	0.916	
SP13 ΔapaH	169.13	0.929	13.64
C1	29.80	0.867	
C1 ΔapaH	69.42	0.918	2.33
BG29	16.95	0.915	
BG29 ΔapaH	552.20	0.899	32.57
BG80	96.49	0.914	
BG80 ΔapaH	422.86	0.945	4.38

Figure S1A

Strain	OD600													
	0	2	4	6	8	10	12	14	16	18	20	22	24	Time (h)
PAO1 pME6032 1	0.01	0.07	0.58	1.54	1.96	2.38	2.67	2.57	2.95	3.27	3.48	3.38	3.22	
PAO1 pME6032 2	0.01	0.09	0.77	2.12	2.40	2.75	3.09	3.43	3.54	3.85	3.85	3.85	3.85	
PAO1 pME6032 3	0.01	0.08	0.67	1.75	2.12	2.50	2.80	2.95	3.18	3.60	3.60	3.70	3.55	
PAO1 pMEapaH 1	0.01	0.08	0.76	1.50	1.94	2.33	2.73	2.80	3.00	3.15	3.40	3.40	3.56	
PAO1 pMEapaH 2	0.01	0.08	0.81	2.00	2.25	2.71	2.87	3.30	3.45	3.90	3.90	3.90	3.60	
PAO1 pMEapaH 3	0.01	0.08	0.79	1.80	2.05	2.50	2.82	3.00	3.20	3.60	3.70	3.70	3.58	
PAO1 ΔapaH pME6032 1	0.01	0.01	0.06	0.54	1.15	1.16	1.30	1.40	1.56	1.55	1.47	1.40	1.35	
PAO1 ΔapaH pME6032 2	0.01	0.00	0.10	1.08	1.55	1.76	1.68	1.60	1.56	1.61	1.65	1.72	1.70	
PAO1 ΔapaH pME6032 3	0.01	0.01	0.09	0.82	1.30	1.50	1.45	1.55	1.56	1.58	1.55	1.56	1.55	
PAO1 ΔapaH pMEapaH 1	0.00	0.10	0.84	1.94	2.39	2.57	2.88	3.07	3.15	3.27	3.60	3.62	3.56	
PAO1 ΔapaH pMEapaH 2	0.00	0.09	0.90	2.00	2.51	2.84	2.60	3.01	3.19	3.50	3.55	3.32	3.28	
PAO1 ΔapaH pMEapaH 3	0.00	0.09	0.88	1.72	2.16	2.45	2.88	2.72	2.89	3.18	3.34	3.49	3.66	

Strain	AVERAGE													
	0	2	4	6	8	10	12	14	16	18	20	22	24	Time (h)
PAO1 pME6032	0.01	0.08	0.67	1.80	2.16	2.54	2.85	2.98	3.22	3.57	3.64	3.64	3.54	
PAO1 pMEapaH	0.01	0.08	0.79	1.77	2.08	2.51	2.81	3.03	3.22	3.55	3.67	3.67	3.58	
PAO1 ΔapaH pME6032	0.01	0.01	0.08	0.81	1.33	1.47	1.48	1.52	1.56	1.58	1.56	1.56	1.53	
PAO1 ΔapaH pMEapaH	0.00	0.09	0.87	1.89	2.35	2.62	2.79	2.93	3.08	3.31	3.50	3.48	3.50	

Strain	SD													
	0	2	4	6	8	10	12	14	16	18	20	22	24	Time (h)
PAO1 pME6032	0.00	0.01	0.10	0.29	0.22	0.19	0.22	0.43	0.30	0.29	0.19	0.24	0.32	
PAO1 pMEapaH	0.00	0.00	0.03	0.25	0.16	0.19	0.07	0.25	0.23	0.38	0.25	0.25	0.02	
PAO1 ΔapaH pME6032	0.00	0.01	0.02	0.27	0.20	0.30	0.19	0.10	0.00	0.03	0.09	0.16	0.18	
PAO1 ΔapaH pMEapaH	0.00	0.00	0.03	0.15	0.18	0.20	0.16	0.19	0.17	0.17	0.14	0.15	0.20	

Figure S1B

Strain	Replicate	Ap4A (pmol/mg of proteins)
PAO1 pME6032	1	7.6
PAO1 pMEapaH	1	1.7
PAO1 ΔapaH pME6032	1	146.7
PAO1 ΔapaH pMEapaH	1	3.8
PAO1 pME6032	2	12.2
PAO1 pMEapaH	2	3.3
PAO1 ΔapaH pME6032	2	128.0
PAO1 ΔapaH pMEapaH	2	3.2
PAO1 pME6032	3	20.6
PAO1 pMEapaH	3	3.4
PAO1 ΔapaH pME6032	3	58.3
PAO1 ΔapaH pMEapaH	3	5.0

	Ap4A (pmol) per mg of protein	
	Average	SD
PAO1 pME6032	13.5	6.60
PAO1 pMEapaH	2.8	0.95
PAO1 ΔapaH pME6032	111.0	46.58
PAO1 ΔapaH pMEapaH	4.0	0.94

Figure S2A

Replicate	Strain	ADP (pmol/mg of proteins)	ATP (pmol/mg of proteins)	GDP (pmol/mg of proteins)	GTP (pmol/mg of proteins)
1	PAO1	948.5	46.4	272.8	39.9
1	PAO1 ΔapaH	754.7	47.0	257.7	42.8
2	PAO1	957.1	41.6	185.4	38.2
2	PAO1 ΔapaH	721.6	41.8	249.5	38.1
3	PAO1	788.8	55.9	221.7	48.9
3	PAO1 ΔapaH	655.9	52.7	198.4	40.6

	Strain	ADP (pmol/mg of proteins)	ATP (pmol/mg of proteins)	GDP (pmol/mg of proteins)	GTP (pmol/mg of proteins)
Average	PAO1	898.1	47.9	226.6	42.3
	PAO1 ΔapaH	710.8	47.2	235.2	40.5
SD	PAO1	77.4	5.9	35.9	4.7
	PAO1 ΔapaH	41.1	4.4	26.2	1.9

	Strain	Levels relative to PAO1			
		ADP	ATP	GDP	GTP
Average	PAO1	1.000	1.000	1.000	1.000
	PAO1 ΔapaH	0.791	0.984	1.038	0.957
SD	PAO1	0.086	0.123	0.158	0.111
	PAO1 ΔapaH	0.046	0.093	0.116	0.045

		pmol/mg of proteins	
Replicate	Strain	ADP+ATP	GDP+GTP
1	PAO1	994.8	312.7
1	PAO1 ΔapaH	801.7	300.5
2	PAO1	998.7	223.5
2	PAO1 ΔapaH	763.5	287.6
3	PAO1	844.7	270.6
3	PAO1 ΔapaH	708.6	239.0

	Strain	ADP+ATP	GDP+GTP
Average	PAO1	946.1	269.0
	PAO1 ΔapaH	757.9	275.7
SD	PAO1	71.7	36.4
	PAO1 ΔapaH	38.2	26.5

Figure S2B

Replicate	Strain	ADP (pmol/mg of proteins)	ATP (pmol/mg of proteins)	GDP (pmol/mg of proteins)	GTP (pmol/mg of proteins)
1	PAO1 pME6032	830.9	40.1	240.4	37.2
1	PAO1 pMEapaH	889.4	44.3	231.1	28.0
1	PAO1 ΔapaH pME6032	625.9	34.9	242.4	32.1
1	PAO1 ΔapaH pMEapaH	842.0	37.8	208.2	25.4
2	PAO1 pME6032	925.1	35.8	224.7	22.3
2	PAO1 pMEapaH	1031.2	44.8	265.6	36.9
2	PAO1 ΔapaH pME6032	699.5	45.9	173.2	25.0
2	PAO1 ΔapaH pMEapaH	826.0	46.0	251.7	21.4
3	PAO1 pME6032	1024.2	56.4	286.2	34.4
3	PAO1 pMEapaH	924.0	56.8	295.7	28.5
3	PAO1 ΔapaH pME6032	620.1	45.6	239.7	30.1
3	PAO1 ΔapaH pMEapaH	881.1	51.5	260.8	36.6

	Strain	ADP (pmol/mg of proteins)	ATP (pmol/mg of proteins)	GDP (pmol/mg of proteins)	GTP (pmol/mg of proteins)
Average	PAO1 pME6032	926.7	44.1	250.4	31.3
	PAO1 pMEapaH	948.2	48.6	264.1	31.1
	PAO1 ΔapaH pME6032	648.5	42.1	218.4	29.1
	PAO1 ΔapaH pMEapaH	849.7	45.1	240.2	27.8
SD	PAO1 pME6032	78.9	8.9	26.1	6.5
	PAO1 pMEapaH	60.4	5.8	26.4	4.1
	PAO1 ΔapaH pME6032	36.2	5.1	32.0	3.0
	PAO1 ΔapaH pMEapaH	23.1	5.6	22.9	6.5

	Strain	Levels relative to PAO1 pME6032			
		ADP	ATP	GDP	GTP
Average	PAO1 pME6032	1.000	1.000	1.000	1.000
	PAO1 pMEapaH	1.023	1.103	1.055	0.995
	PAO1 ΔapaH pME6032	0.700	0.955	0.872	0.930
	PAO1 ΔapaH pMEapaH	0.917	1.023	0.959	0.888
SD	PAO1 pME6032	0.085	0.201	0.104	0.207
	PAO1 pMEapaH	0.065	0.130	0.105	0.130
	PAO1 ΔapaH pME6032	0.039	0.117	0.128	0.096
	PAO1 ΔapaH pMEapaH	0.025	0.127	0.092	0.206

		pmol/mg of proteins	
Replicate	Strain	ADP+ATP	GDP+GTP
1	PAO1 pME6032	871.0	277.6
1	PAO1 pMEapaH	933.7	259.1
1	PAO1 ΔapaH pME6032	660.7	274.5
1	PAO1 ΔapaH pMEapaH	879.9	233.6
2	PAO1 pME6032	960.9	246.9
2	PAO1 pMEapaH	1076.0	302.5
2	PAO1 ΔapaH pME6032	745.4	198.2
2	PAO1 ΔapaH pMEapaH	872.0	273.1
3	PAO1 pME6032	1080.6	320.6
3	PAO1 pMEapaH	980.7	324.2
3	PAO1 ΔapaH pME6032	665.7	269.9
3	PAO1 ΔapaH pMEapaH	932.6	297.4

	Strain	ADP+ATP	GDP+GTP
Average	PAO1 pME6032	970.8	281.7
	PAO1 pMEapaH	996.8	295.3
	PAO1 ΔapaH pME6032	690.6	247.5
	PAO1 ΔapaH pMEapaH	894.8	268.0
SD	PAO1 pME6032	85.9	30.2
	PAO1 pMEapaH	59.2	27.1
	PAO1 ΔapaH pME6032	38.8	34.9
	PAO1 ΔapaH pMEapaH	26.9	26.3

Figure S3A

Strain	C4-HSL (µM)/OD600
PAO1 1	5.81
PAO1 2	4.78
PAO1 3	5.81
PAO1 4	6.18
PAO1 ΔapaH 1	4.57
PAO1 ΔapaH 2	3.39
PAO1 ΔapaH 3	4.14
PAO1 ΔapaH 4	5.05
PAO1 pME6032 1	6.30
PAO1 pME6032 2	6.65
PAO1 pME6032 3	5.31
PAO1 pMEapaH 1	6.11
PAO1 pMEapaH 2	7.45
PAO1 pMEapaH 3	5.25
PAO1 ΔapaH pME6032 1	4.26
PAO1 ΔapaH pME6032 2	4.63
PAO1 ΔapaH pME6032 3	5.19
PAO1 ΔapaH pMEapaH 1	6.86
PAO1 ΔapaH pMEapaH 2	6.15
PAO1 ΔapaH pMEapaH 3	5.50
PAO1 ΔapaH pMEapaH 4	7.25

	C4-HSL (µM)/OD600	
	Average	SD
PAO1	5.65	0.60
PAO1 ΔapaH	4.29	0.71
PAO1 pME6032	6.09	0.70
PAO1 pMEapaH	6.27	1.11
PAO1 ΔapaH pME6032	4.69	0.47
PAO1 ΔapaH pMEapaH	6.44	0.78

Figure S3B

Strain	C12-HSL (μM)/OD600
PAO1 1	7.09
PAO1 2	8.86
PAO1 3	5.31
PAO1 4	5.18
PAO1 ΔapaH 1	6.25
PAO1 ΔapaH 2	7.09
PAO1 ΔapaH 3	8.57
PAO1 ΔapaH 4	8.90
PAO1 pME6032 1	5.91
PAO1 pME6032 2	7.03
PAO1 pME6032 3	8.60
PAO1 pMEapaH 1	6.17
PAO1 pMEapaH 2	7.32
PAO1 pMEapaH 3	8.38
PAO1 ΔapaH pME6032 1	5.95
PAO1 ΔapaH pME6032 2	6.84
PAO1 ΔapaH pME6032 3	8.05
PAO1 ΔapaH pMEapaH 1	7.72
PAO1 ΔapaH pMEapaH 2	9.36
PAO1 ΔapaH pMEapaH 3	6.57
PAO1 ΔapaH pMEapaH 4	6.29

	C12-HSL (μM)/OD600	
	Average	SD
PAO1	6.61	1.74
PAO1 ΔapaH	7.70	1.25
PAO1 pME6032	7.18	1.35
PAO1 pMEapaH	7.29	1.11
PAO1 ΔapaH pME6032	6.95	1.06
PAO1 ΔapaH pMEapaH	7.49	1.39

Figure S4

Strain	FeCl3 (uM)	OD600																							
		0	0.5	1	1.5	2	2.5	3	3.5	4	4.5	5	5.5	6	6.5	7	7.5	8	8.5	9	9.5	10	10.5	11	11.5
PAO1	6	-0.002	-0.002	-0.002	-0.002	-0.001	0.000	0.002	0.004	0.010	0.036	0.057	0.065	0.079	0.095	0.127	0.195	0.304	0.435	0.691	0.773	0.865	0.931	0.990	1.042
	3	0.000	0.000	0.001	0.001	0.002	0.003	0.004	0.007	0.013	0.048	0.055	0.064	0.084	0.105	0.144	0.239	0.343	0.483	0.672	0.774	0.870	0.938	0.986	1.020
	1.5	-0.002	-0.002	-0.001	-0.001	-0.001	0.000	0.002	0.004	0.009	0.049	0.057	0.063	0.089	0.101	0.125	0.211	0.311	0.440	0.549	0.635	0.701	0.750	0.789	0.818
	0.75	-0.002	-0.002	-0.002	-0.001	-0.001	0.000	0.002	0.004	0.010	0.053	0.061	0.066	0.092	0.101	0.122	0.211	0.316	0.381	0.430	0.481	0.513	0.542	0.561	0.580
	0.38	-0.002	-0.001	0.001	0.001	0.003	0.004	0.005	0.007	0.013	0.056	0.062	0.067	0.087	0.088	0.118	0.182	0.240	0.273	0.298	0.333	0.359	0.378	0.393	0.406
	0.19	-0.002	-0.001	0.001	0.001	0.003	0.004	0.005	0.007	0.013	0.048	0.064	0.072	0.089	0.096	0.120	0.139	0.168	0.183	0.204	0.230	0.248	0.263	0.275	0.286
	0.09	-0.002	-0.001	0.001	0.001	0.003	0.004	0.005	0.007	0.013	0.056	0.064	0.076	0.086	0.089	0.090	0.118	0.124	0.137	0.151	0.163	0.174	0.183	0.195	0.206
	0	-0.001	-0.001	-0.001	-0.001	0.000	0.001	0.003	0.006	0.013	0.028	0.037	0.040	0.042	0.048	0.052	0.054	0.061	0.065	0.080	0.083	0.090	0.096	0.099	0.102
ΔapaH	6	0.001	0.001	0.001	0.002	0.002	0.002	0.002	0.005	0.006	0.006	0.007	0.008	0.007	0.016	0.030	0.046	0.059	0.080	0.098	0.133	0.174	0.233	0.328	0.441
	3	0.001	0.001	0.001	0.002	0.002	0.002	0.002	0.005	0.006	0.006	0.007	0.008	0.006	0.009	0.018	0.038	0.052	0.070	0.089	0.108	0.137	0.191	0.258	0.338
	1.5	0.001	0.001	0.001	0.002	0.002	0.002	0.002	0.005	0.006	0.006	0.007	0.008	0.009	0.011	0.017	0.042	0.052	0.061	0.078	0.094	0.120	0.157	0.233	0.321
	0.75	0.001	0.001	0.001	0.002	0.002	0.002	0.002	0.005	0.006	0.006	0.007	0.008	0.009	0.011	0.016	0.028	0.047	0.053	0.061	0.083	0.103	0.123	0.170	0.230
	0.38	0.001	0.001	0.001	0.002	0.002	0.002	0.002	0.005	0.006	0.006	0.007	0.008	0.007	0.016	0.030	0.045	0.060	0.073	0.100	0.114	0.123	0.131	0.130	0.161
	0.19	0.001	0.001	0.001	0.002	0.002	0.002	0.002	0.005	0.006	0.006	0.007	0.008	0.007	0.016	0.029	0.046	0.059	0.070	0.102	0.111	0.119	0.127	0.132	0.137
	0.09	0.001	0.001	0.001	0.002	0.002	0.002	0.002	0.005	0.006	0.006	0.006	0.007	0.007	0.015	0.028	0.045	0.052	0.061	0.062	0.068	0.073	0.078	0.083	0.088
	0	0.001	0.001	0.001	0.002	0.002	0.002	0.002	0.003	0.007	0.008	0.010	0.012	0.013	0.013	0.014	0.014	0.015	0.015	0.015	0.014	0.014	0.014	0.015	0.015

Strain	FeCl3 (uM)	OD600																						
		12	12.5	13	13.5	14	14.5	15	15.5	16	16.5	17	17.5	18	18.5	19	19.5	20	20.5	21	21.5	22	22.5	23
PAO1	6	1.072	1.088	1.104	1.116	1.125	1.135	1.144	1.146	1.149	1.156	1.155	1.153	1.152	1.154	1.152	1.153	1.153	1.153	1.153	1.156	1.158	1.159	1.164
	3	1.050	1.076	1.096	1.114	1.129	1.139	1.146	1.146	1.147	1.149	1.145	1.143	1.141	1.138	1.137	1.134	1.128	1.125	1.123	1.121	1.119	1.118	1.116
	1.5	0.849	0.872	0.888	0.901	0.911	0.919	0.929	0.931	0.940	0.947	0.949	0.953	0.952	0.953	0.949	0.948	0.944	0.939	0.939	0.938	0.936	0.934	0.933
	0.75	0.600	0.617	0.631	0.641	0.646	0.651	0.659	0.660	0.665	0.672	0.672	0.674	0.678	0.679	0.680	0.682	0.683	0.681	0.684	0.686	0.686	0.686	0.689
	0.38	0.421	0.430	0.435	0.441	0.453	0.459	0.467	0.467	0.472	0.477	0.478	0.481	0.483	0.485	0.486	0.489	0.491	0.490	0.492	0.493	0.494	0.495	0.498
	0.19	0.298	0.305	0.312	0.319	0.321	0.324	0.330	0.330	0.334	0.340	0.342	0.344	0.347	0.349	0.350	0.353	0.355	0.354	0.356	0.359	0.361	0.363	0.366
	0.09	0.215	0.223	0.234	0.237	0.240	0.244	0.248	0.250	0.252	0.256	0.256	0.256	0.260	0.261	0.263	0.265	0.266	0.267	0.270	0.273	0.274	0.277	0.279
	0	0.106	0.110	0.113	0.114	0.117	0.120	0.125	0.124	0.126	0.129	0.130	0.132	0.132	0.133	0.134	0.134	0.134	0.135	0.134	0.135	0.135	0.137	0.134
ΔapaH	6	0.545	0.644	0.739	0.813	0.873	0.919	0.959	0.984	1.007	1.031	1.048	1.063	1.077	1.090	1.100	1.108	1.116	1.124	1.128	1.133	1.137	1.137	1.143
	3	0.439	0.547	0.646	0.714	0.765	0.801	0.839	0.866	0.890	0.913	0.928	0.938	0.950	0.955	0.957	0.956	0.956	0.953	0.951	0.950	0.945	0.945	0.943
	1.5	0.405	0.473	0.531	0.571	0.599	0.624	0.648	0.663	0.675	0.691	0.697	0.704	0.710	0.713	0.718	0.718	0.719	0.716	0.716	0.713	0.712	0.710	0.709
	0.75	0.296	0.333	0.357	0.387	0.412	0.434	0.451	0.461	0.471	0.483	0.485	0.489	0.487	0.489	0.491	0.494	0.496	0.492	0.496	0.498	0.498	0.497	0.500
	0.38	0.187	0.208	0.227	0.237	0.258	0.269	0.284	0.290	0.300	0.308	0.311	0.315	0.325	0.319	0.319	0.320	0.324	0.323	0.327	0.330	0.333	0.341	0.341
	0.19	0.142	0.147	0.151	0.155	0.158	0.160	0.161	0.163	0.165	0.167	0.170	0.171	0.171	0.173	0.173	0.174	0.175	0.175	0.176	0.177	0.178	0.178	0.179
	0.09	0.093	0.095	0.100	0.102	0.104	0.106	0.108	0.109	0.111	0.112	0.113	0.115	0.116	0.116	0.117	0.119	0.119	0.120	0.120	0.121	0.121	0.120	0.120
	0	0.015	0.017	0.018	0.018	0.018	0.019	0.019	0.020	0.020	0.021	0.021	0.021	0.021	0.022	0.022	0.022	0.023	0.024	0.025	0.026	0.027	0.027	0.028

Time (h)

Figure S5A

Replicate 1	CFU/mL					
Strain	0	30	60	120	240	Time (min)
PAO1	1.6E+09	1.6E+09	3.1E+08			
ΔapaH	6.0E+08	1.7E+09	2.3E+08			

Replicate 2	CFU/mL					
Strain	0	30	60	120	240	Time (min)
PAO1	2.3E+09	1.6E+09	9.0E+08	3.6E+08	2.3E+07	
ΔapaH	1.8E+09	1.6E+09	3.5E+08	4.5E+06	5.1E+04	

Replicate 3	CFU/mL					
Strain	0	30	60	120	240	Time (min)
PAO1	1.9E+09	8.0E+08	2.9E+08	4.5E+07	3.4E+06	
ΔapaH	2.3E+09	5.0E+08	1.4E+08	1.5E+06	2.0E+04	

Replicate 4	CFU/mL					
Strain	0	30	60	120	240	Time (min)
PAO1	1.4E+09	1.3E+09	3.8E+08	2.0E+08	2.0E+07	
ΔapaH	1.8E+09	1.7E+09	2.6E+08	8.3E+06	3.1E+04	

AVERAGE	CFU/mL					
Strain	0	30	60	120	240	Time (min)
PAO1	1.8E+09	1.3E+09	4.7E+08	2.0E+08	1.6E+07	
ΔapaH	1.6E+09	1.4E+09	2.4E+08	4.8E+06	3.4E+04	

SD	CFU/mL					
Strain	0	30	60	120	240	Time (min)
PAO1	3.9E+08	3.7E+08	2.9E+08	1.6E+08	1.1E+07	
ΔapaH	7.0E+08	5.8E+08	8.6E+07	3.4E+06	1.6E+04	

Figure S5B

Replicate 1	CFU/mL					
Strain	0	30	60	120	240	Time (min)
PAO1 pME6032	1.1E+09	9.0E+08	3.2E+08			
PAO1 pMEapaH	1.6E+09	1.2E+09	2.9E+08			
ΔapaH pME6032	9.0E+08	1.5E+09	2.7E+08			
ΔapaH pMEapaH	8.0E+08	6.5E+08	3.8E+08			

AVERAGE	CFU/mL					
Strain	0	30	60	120	240	Time (min)
PAO1 pME6032	1.4E+09	1.2E+09	3.8E+08	1.2E+08	6.5E+06	
PAO1 pMEapaH	1.5E+09	1.5E+09	4.8E+08	8.9E+07	2.0E+06	
ΔapaH pME6032	1.2E+09	1.3E+09	1.9E+08	3.3E+06	2.6E+04	
ΔapaH pMEapaH	1.6E+09	1.0E+09	3.1E+08	1.3E+08	3.9E+06	

Replicate 2	CFU/mL					
Strain	0	30	60	120	240	Time (min)
PAO1 pME6032	1.9E+09	1.8E+09	6.0E+08	2.3E+08	9.7E+06	
PAO1 pMEapaH	1.9E+09	2.4E+09	8.5E+08	1.2E+08	3.0E+06	
ΔapaH pME6032	1.4E+09	1.9E+09	2.0E+08	3.5E+06	3.9E+04	
ΔapaH pMEapaH	2.3E+09	1.5E+09	2.3E+08	1.7E+08	5.8E+06	

SD	CFU/mL					
Strain	0	30	60	120	240	Time (min)
PAO1 pME6032	5.4E+08	5.3E+08	1.7E+08	1.0E+08	5.4E+06	
PAO1 pMEapaH	3.6E+08	6.5E+08	2.7E+08	4.5E+07	1.5E+06	
ΔapaH pME6032	3.0E+08	4.8E+08	6.4E+07	2.8E+06	1.6E+04	
ΔapaH pMEapaH	7.1E+08	3.9E+08	1.3E+08	8.3E+07	1.9E+06	

Replicate 3	CFU/mL					
Strain	0	30	60	120	240	Time (min)
PAO1 pME6032	1.9E+09	6.0E+08	2.0E+08	2.5E+07	2.0E+05	
PAO1 pMEapaH	1.7E+09	1.5E+09	2.8E+08	3.8E+07	2.8E+05	
ΔapaH pME6032	1.6E+09	7.0E+08	1.1E+08	3.8E+05	7.8E+03	
ΔapaH pMEapaH	2.1E+09	7.5E+08	1.8E+08	3.1E+07	2.1E+06	

Replicate 4	CFU/mL					
Strain	0	30	60	120	240	Time (min)
PAO1 pME6032	8.5E+08	1.4E+09	4.1E+08	1.1E+08	9.5E+06	
PAO1 pMEapaH	1.0E+09	9.0E+08	5.0E+08	1.2E+08	2.7E+06	
ΔapaH pME6032	1.1E+09	1.4E+09	2.0E+08	5.9E+06	3.1E+04	
ΔapaH pMEapaH	1.2E+09	1.1E+09	4.5E+08	1.8E+08	3.7E+06	

Figure S6A

Strain	OD400/OD600
PAO1 pME6032 1	1.31
PAO1 pME6032 2	1.16
PAO1 pME6032 3	1.06
PAO1 pMEapaH 1	1.13
PAO1 pMEapaH 2	1.32
PAO1 pMEapaH 3	1.56
PAO1 ΔapaH pME6032 1	0.04
PAO1 ΔapaH pME6032 2	0.12
PAO1 ΔapaH pME6032 3	0.06
PAO1 ΔapaH pMEapaH 1	1.14
PAO1 ΔapaH pMEapaH 2	1.42
PAO1 ΔapaH pMEapaH 3	1.32

	OD400/OD600	
	Average	SD
PAO1 pME6032	1.18	0.13
PAO1 pMEapaH	1.34	0.21
PAO1 ΔapaH pME6032	0.07	0.04
PAO1 ΔapaH pMEapaH	1.29	0.14

Figure S6B

Strain	OD495/OD600
PAO1 pME6032 1	0.11
PAO1 pME6032 2	0.09
PAO1 pME6032 3	0.13
PAO1 pMEapaH 1	0.12
PAO1 pMEapaH 2	0.13
PAO1 pMEapaH 3	0.11
PAO1 ΔapaH pME6032 1	0.01
PAO1 ΔapaH pME6032 2	0.00
PAO1 ΔapaH pME6032 3	0.01
PAO1 ΔapaH pMEapaH 1	0.08
PAO1 ΔapaH pMEapaH 2	0.11
PAO1 ΔapaH pMEapaH 3	0.09

	OD495/OD600	
	Average	SD
PAO1 pME6032	0.11	0.02
PAO1 pMEapaH	0.12	0.01
PAO1 ΔapaH pME6032	0.01	0.00
PAO1 ΔapaH pMEapaH	0.09	0.01

Figure S6C

Strain	PQS (µM)/OD600
PAO1 pME6032 1	8.10
PAO1 pME6032 2	10.23
PAO1 pME6032 3	7.67
PAO1 pMEapaH 1	7.86
PAO1 pMEapaH 2	10.64
PAO1 pMEapaH 3	8.36
PAO1 ΔapaH pME6032 1	0.71
PAO1 ΔapaH pME6032 2	1.37
PAO1 ΔapaH pME6032 3	1.18
PAO1 ΔapaH pMEapaH 1	6.10
PAO1 ΔapaH pMEapaH 2	9.39
PAO1 ΔapaH pMEapaH 3	10.74
PAO1 ΔapaH pMEapaH 4	7.93

	PQS (µM)/OD600	
	Average	SD
PAO1 pME6032	8.67	1.37
PAO1 pMEapaH	8.95	1.48
PAO1 ΔapaH pME6032	1.09	0.34
PAO1 ΔapaH pMEapaH	8.54	1.99

Figure S6D

Replicate 1	OD600													
	0	2	4	6	8	10	12	14	16	18	20	22	24	Time (h)
PAO1 pME6032	0.010	0.016	0.084	0.224	0.258	0.275	0.300	0.308	0.319	0.351	0.335	0.325	0.365	
PAO1 pME6032 + Fe	0.010	0.020	0.216	1.042	1.673	1.850	1.940	1.964	2.020	2.180	2.385	2.302	1.981	
PAO1 pMapaH	0.010	0.016	0.080	0.214	0.248	0.200	0.298	0.314	0.344	0.338	0.315	0.345	0.356	
PAO1 pMapaH + Fe	0.010	0.020	0.236	0.978	1.694	1.748	1.700	1.964	2.026	2.080	2.340	2.300	1.900	
ΔapaH pME6032	0.010	0.016	0.052	0.080	0.090	0.098	0.100	0.110	0.116	0.116	0.115	0.130	0.136	
ΔapaH pME6032 + Fe	0.010	0.017	0.064	0.309	1.205	1.674	1.821	1.869	1.950	2.076	2.205	2.189	1.935	
ΔapaH pMEapaH	0.001	0.012	0.112	0.288	0.296	0.312	0.304	0.316	0.352	0.368	0.320	0.348	0.356	
ΔapaH pMEapaH + Fe	0.001	0.015	0.132	0.744	1.640	1.818	1.886	2.128	2.544	2.388	2.160	2.420	2.414	

Replicate 2	OD600												
	0	2	4	6	8	10	12	14	16	18	20	22	24
PAO1 pME6032	0.010	0.015	0.080	0.218	0.248	0.266	0.290	0.298	0.311	0.359	0.324	0.335	0.379
PAO1 pME6032 + Fe	0.010	0.019	0.205	1.054	1.769	1.893	1.853	1.866	1.919	2.071	2.280	2.169	1.940
PAO1 pMapaH	0.010	0.015	0.076	0.204	0.240	0.190	0.290	0.305	0.333	0.340	0.314	0.343	0.346
PAO1 pMapaH + Fe	0.010	0.019	0.224	0.940	1.640	1.772	1.713	1.885	1.942	1.965	2.212	2.185	1.805
ΔapaH pME6032	0.010	0.015	0.049	0.076	0.086	0.093	0.095	0.105	0.110	0.110	0.109	0.124	0.129
ΔapaH pME6032 + Fe	0.010	0.020	0.059	0.284	1.163	1.695	1.749	1.771	1.874	1.972	2.114	2.104	1.862
ΔapaH pMEapaH	0.001	0.005	0.079	0.188	0.232	0.268	0.321	0.338	0.344	0.368	0.388	0.396	0.385
ΔapaH pMEapaH + Fe	0.001	0.006	0.088	0.824	1.992	2.132	2.296	2.176	2.144	2.392	2.504	2.448	2.480

Replicate 3	OD600												
	0	2	4	6	8	10	12	14	16	18	20	22	24
PAO1 pME6032	0.010	0.018	0.092	0.226	0.270	0.294	0.320	0.329	0.338	0.368	0.358	0.358	0.387
PAO1 pME6032 + Fe	0.010	0.022	0.238	1.087	1.954	1.905	1.994	2.060	2.122	2.298	2.530	2.508	2.175
PAO1 pMapaH	0.010	0.018	0.088	0.228	0.265	0.220	0.315	0.298	0.324	0.355	0.337	0.372	0.378
PAO1 pMapaH + Fe	0.010	0.022	0.250	1.065	1.765	1.878	1.841	2.082	2.244	2.290	2.546	2.530	2.090
ΔapaH pME6032	0.010	0.018	0.057	0.088	0.099	0.108	0.110	0.121	0.128	0.128	0.127	0.143	0.150
ΔapaH pME6032 + Fe	0.010	0.022	0.075	0.321	1.289	1.742	1.950	2.058	2.184	2.184	2.368	2.384	2.102
ΔapaH pMEapaH	0.001	0.004	0.086	0.204	0.316	0.326	0.312	0.352	0.348	0.372	0.364	0.404	0.425
ΔapaH pMEapaH + Fe	0.001	0.008	0.112	0.804	2.116	2.364	2.404	2.352	2.084	2.184	2.428	2.552	2.510

	AVERAGE													
	0	2	4	6	8	10	12	14	16	18	20	22	24	Time (h)
PAO1 pME6032	0.010	0.016	0.085	0.223	0.258	0.278	0.303	0.311	0.323	0.359	0.339	0.339	0.377	
PAO1 pME6032 + Fe	0.010	0.020	0.220	1.061	1.799	1.883	1.929	1.963	2.020	2.183	2.398	2.326	2.032	
PAO1 pMapaH	0.010	0.016	0.081	0.215	0.251	0.203	0.301	0.306	0.334	0.345	0.322	0.353	0.360	
PAO1 pMapaH + Fe	0.010	0.020	0.237	0.994	1.700	1.799	1.751	1.977	2.071	2.112	2.366	2.338	1.932	
ΔapaH pME6032	0.010	0.016	0.053	0.081	0.092	0.100	0.102	0.112	0.118	0.118	0.117	0.132	0.138	
ΔapaH pME6032 + Fe	0.010	0.020	0.066	0.305	1.219	1.704	1.840	1.899	2.003	2.077	2.229	2.226	1.966	
ΔapaH pMEapaH	0.001	0.007	0.092	0.227	0.281	0.302	0.312	0.335	0.348	0.369	0.357	0.383	0.389	
ΔapaH pMEapaH + Fe	0.001	0.010	0.111	0.791	1.916	2.105	2.195	2.219	2.257	2.321	2.364	2.473	2.468	

	SD													
	0	2	4	6	8	10	12	14	16	18	20	22	24	Time (h)
PAO1 pME6032	0.00	0.00	0.01	0.00	0.01	0.01	0.02	0.02	0.01	0.01	0.02	0.02	0.01	
PAO1 pME6032 + Fe	0.00	0.00	0.02	0.02	0.14	0.03	0.07	0.10	0.10	0.11	0.13	0.17	0.13	
PAO1 pMapaH	0.00	0.00	0.01	0.01	0.01	0.02	0.01	0.01	0.01	0.01	0.01	0.02	0.02	
PAO1 pMapaH + Fe	0.00	0.00	0.01	0.06	0.06	0.07	0.08	0.10	0.16	0.16	0.17	0.18	0.15	
ΔapaH pME6032	0.00	0.00	0.00	0.01	0.01	0.01	0.01	0.01	0.01	0.01	0.01	0.01	0.01	
ΔapaH pME6032 + Fe	0.00	0.00	0.01	0.02	0.06	0.03	0.10	0.15	0.16	0.11	0.13	0.14	0.12	
ΔapaH pMEapaH	0.00	0.00	0.02	0.05	0.04	0.03	0.01	0.02	0.00	0.00	0.03	0.03	0.03	
ΔapaH pMEapaH + Fe	0.00	0.00	0.02	0.04	0.25	0.27	0.27	0.12	0.25	0.12	0.18	0.07	0.05	

Figure S6E

Strain	OD405/OD600	
	CAA	CAA+Fe
PAO1 pME6032 1	4.78	0.08
PAO1 pME6032 2	3.96	0.08
PAO1 pME6032 3	4.34	0.08
PAO1 pMEapaH 1	4.67	0.08
PAO1 pMEapaH 2	3.78	0.08
PAO1 pMEapaH 3	3.19	0.08
PAO1 ΔapaH pME6032 1	1.26	0.11
PAO1 ΔapaH pME6032 2	0.94	0.09
PAO1 ΔapaH pME6032 3	0.98	0.10
PAO1 ΔapaH pMEapaH 1	3.52	0.09
PAO1 ΔapaH pMEapaH 2	4.41	0.21
PAO1 ΔapaH pMEapaH 3	3.79	0.11
PAO1 ΔapaH pMEapaH 4	4.16	0.02
PAO1 ΔapaH pMEapaH 5	3.20	0.04
PAO1 ΔapaH pMEapaH 6	2.74	0.04

	OD405/OD600			
	Average		SD	
	CAA	CAA+Fe	CAA	CAA+Fe
PAO1 pME6032	4.36	0.08	0.41	0.00
PAO1 pMEapaH	3.88	0.08	0.75	0.00
PAO1 ΔapaH pME6032	1.06	0.10	0.18	0.01
PAO1 ΔapaH pMEapaH	3.64	0.08	0.62	0.07

Figure S7

Time (h)	PAO1	ΔapaH
20	1	1
20	1	
20	1	
20	1	
20		
24	1	
24	1	
24	1	
24	1	
24	1	
24	1	
24	1	
24	1	
24	1	
24	1	
24	1	
40		1
40		1
40	1	1
40		1
40		1
40		1
40		1
40		1
40		1
40		1
40		1
40		1
48		1
48		1
64		1

Figure S8A

Replicate 1	Relative mRNA levels				
	prpL	pvdD	aprA	lasA	lasB
PAO1 pME6032	1	1	1	1	1
PAO1 pMEapaH	2.08	1.22	1.28	0.91	1.05

Replicate 2	Relative mRNA levels				
	prpL	pvdD	aprA	lasA	lasB
PAO1 pME6032	1	1	1	1	1
PAO1 pMEapaH	2.17	1.31	1.36	1.22	1.38

Replicate 3	Relative mRNA levels				
	prpL	pvdD	aprA	lasA	lasB
PAO1 pME6032	1	1	1	1	1
PAO1 pMEapaH	2.26	1.37	1.42	1.51	1.72

Average	Relative mRNA levels				
	prpL	pvdD	aprA	lasA	lasB
PAO1 pME6032	1	1	1	1	1
PAO1 pMEapaH	2.17	1.30	1.35	1.21	1.39

SD	Relative mRNA levels				
	prpL	pvdD	aprA	lasA	lasB
PAO1 pME6032					
PAO1 pMEapaH	0.09	0.07	0.07	0.30	0.34

Figure S8B

Strain	Replicate	No. of CFUs in 1 mg of lettuce
PAO1 pME6032	1	2.70E+06
	2	1.10E+07
	3	1.90E+07
	4	7.10E+06
	5	1.50E+07
PAO1 pMEapaH	1	4.20E+06
	2	5.10E+06
	3	3.40E+06
	4	8.50E+06
	5	1.00E+07

Figure S8C

PAO1 pME6032	
Infecting dose (CFUs)	Survival
358.75	0
280	0
116.833	0
35.875	0
28	0
11.683	0
3.588	0.2
3.505	0.2
2.8	0.3
1.168	0.4
0.359	0.6
0.351	0.9
0.28	0.7
0.117	0.9
0.035	1

PAO1 pMEapaH	
Infecting dose (CFUs)	Survival
207.5	0
140	0
81.333	0
20.75	0
14.8	0.1
8.133	0.1
2.44	0.2
2.075	0.1
1.48	0.2
0.813	0.4
0.244	0.5
0.208	0.4
0.148	0.7
0.081	1

Figure S9

		OD600	
		WT	ΔapaH
Replicate 1	PA14	2.75	2.12
	TR1	2.35	2.37
	BG29	2.21	2.22
	BG80	1.77	1.78
	C1	2.96	2.30
	SP13	2.18	2.27
Replicate 2	PA14	1.83	2.33
	TR1	2.86	2.70
	BG29	2.08	2.12
	BG80	1.36	1.55
	C1	3.40	2.61
	SP13	2.72	2.28
Replicate 3	PA14	2.62	2.18
	TR1	2.72	2.16
	BG29	2.10	2.35
	BG80	1.51	1.50
	C1	3.19	2.78
	SP13	2.24	2.22

	Average		SD	
	WT	ΔapaH	WT	ΔapaH
PA14	2.40	2.21	0.50	0.11
TR1	2.65	2.41	0.26	0.27
BG29	2.13	2.23	0.07	0.12
BG80	1.55	1.61	0.21	0.15
C1	3.18	2.56	0.22	0.24
SP13	2.38	2.26	0.30	0.03

Figure S10

PA14	
Infecting dose (CFUs)	Survival
350	0
143	0
58.25	0
35	0
14.3	0
5.8	0.1
4.3	0
3.5	0.2
1.4	0.6
1.1	0.4
0.58	0.6
0.43	0.7
0.35	1
0.14	0.9
0.12	1

PA14 ΔapaH	
Infecting dose (CFUs)	Survival
173	0
91	0
58	0
17	0
9.1	0.1
5.8	0.2
5.2	0.6
1.7	0.7
0.91	1
0.58	0.7
0.52	0.9
0.17	1

C1	
Infecting dose (CFUs)	Survival
1550	0
960	0
324	0
155	0
96	0
60	0.1
32	0
16	0.3
9.6	0.1
6.0	0.5
3.2	0.4
1.8	0.8
1.6	0.2
0.96	0.8
0.60	0.9
0.32	0.8
0.18	1
0.16	0.9

C1 ΔapaH	
Infecting dose (CFUs)	Survival
2633	0
750	0
263	0
75	0.1
60	0.3
26	0
7.5	0.5
6.0	0.7
2.6	0.5
1.8	0.8
0.75	1
0.60	0.9
0.26	1
0.18	0.9

SP13	
Infecting dose (CFUs)	Survival
237	0
86	0
53	0
24	0
8.6	0.3
5.3	0.1
2.4	0.7
1.6	0.5
0.86	0.9
0.53	1
0.24	0.8
0.16	0.9

SP13 ΔapaH	
Infecting dose (CFUs)	Survival
1008	0
630	0
239	0.1
101	0
63	0.2
24	0.5
10	0.5
6.3	0.7
3.0	0.9
2.4	0.6
1.0	0.7
0.63	0.8
0.30	1
0.24	0.9
0.10	1

TR1	
Infecting dose (CFUs)	Survival
247	0
181	0
45	0
25	0
18	0
5.4	0.4
4.5	0.3
2.5	0.6
1.8	0.9
0.54	0.6
0.45	1
0.25	0.8
0.18	0.9
0.03	1

TR1 ΔapaH	
Infecting dose (CFUs)	Survival
399	0
114	0
85	0
40	0
11	0
8.5	1
4.0	0
3.4	1
1.1	1
0.85	1
0.40	1
0.34	1
0.11	1

BG29	
Infecting dose (CFUs)	Survival
560	0
97.5	0
56	0
12.5	0.1
9.75	0.3
5.6	0.1
2.925	0.8
1.25	0.6
0.975	0.9
0.56	0.8
0.293	1
0.125	1
0.098	1

BG29 ΔapaH	
Infecting dose (CFUs)	Survival
7400	0
4200	0
420	0.2
102	0.3
74	0.2
42	0.4
10.2	0.9
7.4	0.8
4.2	0.5
3.07	0.9
1.02	1
0.74	0.8
0.42	0.9

BG80	
Infecting dose (CFUs)	Survival
6775	0
678	0
190	0
90	0.2
68	0.1
19	0.2
9.0	0.7
6.8	0.4
2.7	0.8
1.9	0.7
0.90	1
0.68	0.7
0.27	1
0.19	0.9

BG80 ΔapaH	
Infecting dose (CFUs)	Survival
3283	0
1600	0
328	0.1
160	0.2
90	0.4
33	0.7
16	0.8
9.0	0.9
3.3	0.7
2.7	1
1.6	0.9
0.90	1
0.33	0.8
0.27	1
0.16	1

Table S2

	H_2O_2 MIC (mM)			
	Replicate 1	Replicate 2	Replicate 3	Mode
PAO1	1	2	2	2
ΔapaH	2	2	1	2

	Paraquat MIC (mM)			
	Replicate 1	Replicate 2	Replicate 3	Mode
PAO1	0.5	1	0.5	0.5
ΔapaH	0.5	0.5	0.5	0.5
